# Image Encryption Algorithm Based on a Novel 2D Folded Sine-Logistic Map and Concentric-Ring Model

**DOI:** 10.3390/e28080863

**Published:** 2026-08-01

**Authors:** Meixuan Huang, Shaobao Wu, Zhihua Wu, Xiaoqiang Zhang

**Affiliations:** 1 School of Information and Electronic Engineering, Liming Vocational University, Quanzhou 362000, China; 2 School of Information and Control Engineering, China University of Mining and Technology, Xuzhou 221116, China

**Keywords:** image encryption, concentric-ring permutation, enhanced hybrid diffusion, 4-bit nibble-swap

## Abstract

Digital images, as a widely used multimedia carrier, are vulnerable to various security risks in open network environments. To enhance the security of digital images during storage and transmission, this paper proposes an image encryption algorithm based on a 2D folded sine-logistic map. First, the user master key and the SHA-256 hash of the plaintext image are combined to generate plaintext-related chaotic sequences. Second, a concentric-ring permutation is performed through three-ring partition, cyclic shifting, and route regrouping, with an additional channel crossover for RGB images to enhance inter-channel coupling. Finally, an enhanced hybrid diffusion mechanism with 4-bit nibble-swap perturbation is applied to strengthen pixel-value diffusion and local nonlinearity. Experimental results show that the average ciphertext entropy reaches 7.999, the adjacent-pixel correlation coefficient is reduced to 0.000016, and the NPCR/UACI values are close to their theoretical ideal levels. Combined with comprehensive security analyses, these findings demonstrate that the proposed scheme suppresses statistical characteristics and pixel correlations, resists brute-force, statistical, and differential attacks, and provides an efficient and secure solution for grayscale and color image transmission.

## 1. Introduction

With the rapid development of multimedia communication, cloud services, and intelligent sensing systems, digital images have become one of the most important carriers of information in modern networks [1,2,3]. Compared with textual data, images usually contain much richer visual content and are widely used in fields such as telemedicine, remote sensing, industrial inspection, and social media transmission [4,5,6]. However, due to the openness of communication channels and storage environments, image data are vulnerable to unauthorized access, tampering, and malicious interception during transmission and storage [7,8,9]. Therefore, the development of secure and efficient image encryption schemes has become an important research topic in information security [10,11,12].

Traditional text-oriented encryption algorithms, such as AES and DES, can in principle be applied to image protection. Nevertheless, image data are typically characterized by large data volume, high pixel redundancy, and strong correlation among adjacent pixels [13,14,15]. Therefore, directly applying traditional ciphers may not always be the most efficient or structurally suitable solution for image-oriented secure transmission [16,17,18]. In recent years, chaotic image encryption has attracted extensive attention due to the intrinsic properties of chaotic systems, including sensitivity to initial conditions, ergodicity, pseudo-randomness, and parameter dependence [19,20,21]. These properties are highly consistent with the basic requirements of secure image encryption, such as confusion, diffusion, and key sensitivity.

Consequently, numerous chaos-based image encryption methods have been developed, generally combining pixel permutation and value diffusion to conceal statistical characteristics and improve resistance to cryptanalytic attacks. Yang et al. [22] proposed a multi-image compressive encryption scheme driven by a dynamic delayed-feedback chaotic system, in which synchronized fractal diffusion was introduced to enhance inter-image dependency and diffusion complexity. Liu et al. [23] designed a directional shift transformation based on an extended chaotic map to achieve effective spatial redistribution among multiple images. Li et al. [24] constructed a nonlinear-feedback-driven 3D hyperchaotic encryption framework, where the feedback mechanism improved the randomness of chaotic sequences and strengthened the confusion–diffusion process. Jackson and Perumal [25] developed a medical image encryption technique using an inverse cosine chaotic map, combining Josephus block scrambling, global Zigzag permutation, and adaptive diffusion to improve robustness.

Further studies have explored more complex scrambling structures and cross-image diffusion mechanisms. Yang et al. [26] proposed a facial image encryption method based on spatiotemporal chaos with dynamic coefficients, in which a fractal scrambling matrix was used to enhance structural complexity. Zhou et al. [27] introduced a two-dimensional hyperchaotic model and cyclic-shift DNA coding for multi-image encryption, improving both pixel-level permutation and diffusion sensitivity. Lu et al. [28] designed a multiple-image encryption algorithm based on a new 3D hyperchaotic map and a Whac-A-Mole-inspired scrambling model, which increased the randomness of cross-image pixel migration. Gao and Teng [29] proposed a block space jump scrambling strategy combined with dynamic sliding queue diffusion, effectively enhancing global scrambling capability and inter-pixel dependency. Song et al. [30] proposed a batch image encryption method using cross-image permutation and diffusion, in which pixels from different images are jointly rearranged and diffused to strengthen inter-image dependency and enhance resistance to statistical and differential attacks.

Despite these advantages, many existing chaotic image encryption schemes still suffer from several limitations. First, some low-dimensional chaotic maps exhibit limited dynamical complexity, narrow chaotic parameter intervals, or degraded randomness under finite-precision digital implementation, which may weaken their practical security performance [31,32]. Second, in the permutation stage, many schemes rely on simple row-column shuffling, block scrambling, or single-path sorting strategies. Although these methods can disturb pixel positions to a certain extent, their spatial routing capability and structural flexibility may be insufficient for highly correlated image contents [33,34]. In particular, purely local or regular permutation mechanisms may fail to fully exploit global spatial redistribution, thereby reducing the effectiveness of the subsequent diffusion stage. Third, in the diffusion stage, some methods employ relatively simple one-way propagation or locally coupled updates, resulting in insufficient global dependency and limited differential sensitivity. By contrast, some highly nonlinear diffusion structures significantly increase computational burden, which affects practical efficiency [35,36,37]. In addition, if the key-generation process is not sufficiently associated with the plaintext, the encryption system may become more vulnerable to known-plaintext or chosen-plaintext attacks [38].

To address these issues, a new chaotic image encryption scheme is proposed by integrating a two-dimensional folded sine-logistic map (2D-FSLM), a concentric-ring permutation mechanism, and an enhanced hybrid diffusion structure. The proposed 2D-FSLM is constructed by introducing folded coupling into a two-dimensional chaotic framework, thereby improving dynamical complexity and providing richer pseudo-random sequences for encryption. On this basis, a concentric-ring permutation strategy is developed for spatial scrambling. Compared with conventional linear or block-based rearrangement, this structure provides a more flexible routing pattern for pixel migration and is more effective in disrupting the inherent spatial correlation of images.

An enhanced hybrid diffusion mechanism is further designed to modify pixel values after permutation. Different from conventional one-way diffusion, the proposed diffusion structure combines forward diffusion, backward diffusion, forward mixing, and reverse compensation, so that local perturbations can propagate globally throughout the entire image sequence. In addition, plaintext-related information is incorporated into session-key generation, enabling the scheme to achieve stronger key sensitivity and one-image-one-key characteristics. Therefore, the proposed encryption framework integrates improved chaotic dynamics, structured permutation, and strengthened diffusion into a unified system, aiming to achieve a favorable balance between security and computational efficiency. The main contributions of this paper can be summarized as follows.

(1) A novel two-dimensional folded sine-logistic map is developed to generate chaotic sequences with improved dynamical characteristics for image encryption.
(2) A concentric-ring model is constructed to enhance spatial scrambling capability through ring partition, ring-wise ordering, cyclic shifting, and balanced route regrouping, and an additional channel permutation operation is incorporated for RGB images.
(3) An enhanced hybrid diffusion mechanism integrating forward diffusion, backward diffusion, forward mixing, and reverse compensation is designed to improve global inter-pixel dependency, diffusion nonlinearity, and resistance to differential attacks.

The remainder of this paper is organized as follows. Section 2 describes the preliminaries, including the theoretical basis of the concentric-ring model and the construction of the proposed 2D-FSLM, followed by analyses of its chaotic properties and randomness performance. Section 3 presents the proposed image encryption algorithm, including key-generation strategy, concentric-ring model, diffusion mask and seed generation, and enhanced hybrid diffusion. Section 4 reports the experimental results and algorithm analyses. Finally, Section 5 concludes the paper and presents the outlook.

## 2. Preliminaries

### 2.1. Designed Concentric-Ring Model

**Figure 1:**
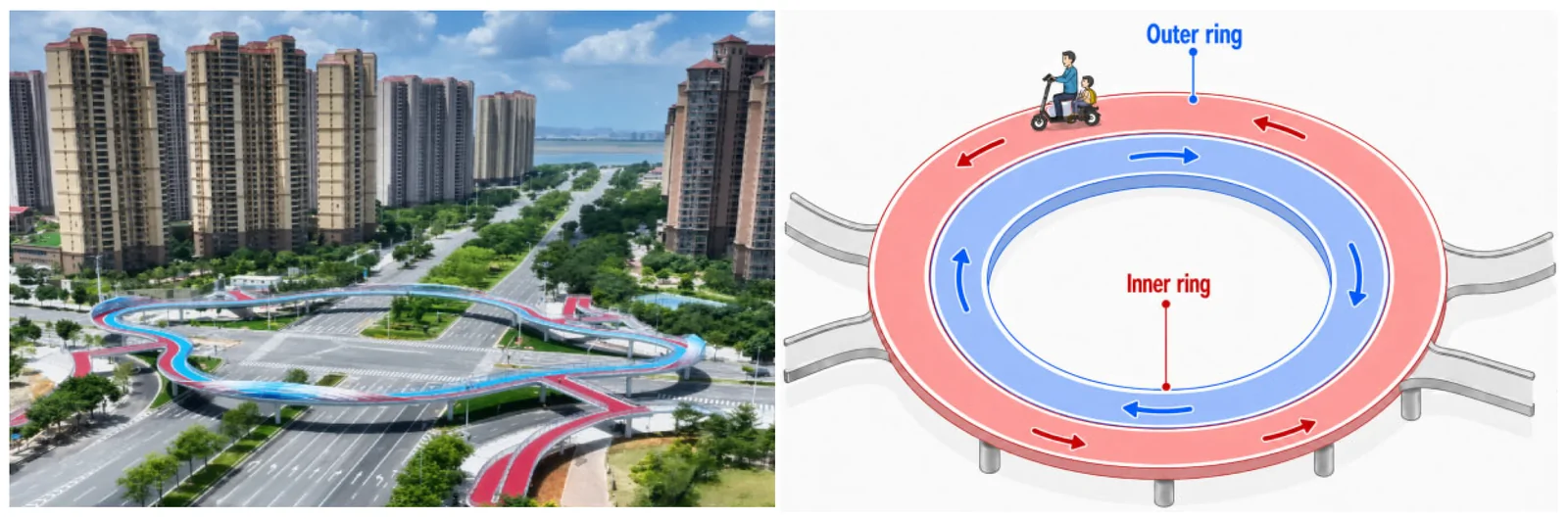
Inspiration Source of the Concentric-Ring Model.

There is a petal-shaped circular overpass in Quanzhou, China, whose circulation and routing behavior provides a natural analogy for pixel rearrangement, as shown in Figure 1. In such a structure, moving objects enter from different entrances, circulate along ring-like paths, and leave through different exits after route-guided redistribution. To avoid possible collisions, traffic on the outer ring proceeds in the counterclockwise direction, whereas traffic on the inner ring moves in the clockwise direction. Motivated by this behavior, the proposed permutation mechanism organizes pixels into concentric regions and performs controlled cross-region rearrangement, thereby improving permutation flexibility and spatial scrambling capability.

### 2.2. Proposed 2D-FSLM

#### 2.2.1. Construction of the Proposed Map

A two-dimensional folded sine-logistic map is constructed to generate chaotic sequences for the subsequent permutation and diffusion stages. Let $x_{n},y_{n}\in [0,1)$ denote the two state variables at the *n*-th iteration. The proposed 2D-FSLM is defined as

(1) $$\left\{\begin{array}{l} x_{n+1}=\mathrm{mod}(T(u_{n})+0.19v_{n},1)\\ y_{n+1}=\mathrm{mod}(T(v_{n})+0.17x_{n+1},1) \end{array},\right.$$

where

(2) $$\left\{\begin{array}{c} u_{n}=\mathrm{mod}(x_{n}+\mu \sin(2\pi y_{n})+\beta \sin(2\pi (x_{n}+y_{n})+0.31y_{n}^{2},1)\\ v_{n}=\mathrm{mod}(y_{n}+\mu \cos(2\pi x_{n})+\beta \cos(2\pi (x_{n}-y_{n})+0.27x_{n}^{2},1) \end{array},\right.$$

μ and β are control parameters, mod(•,1) denotes the modulo operation that maps its input into [0, 1), and T(•) is a folding operator defined by the tent transformation

(3) $$T(z)=1-2\left|z-0.5\right|.$$

In this structure, the sine-cosine coupling introduces cross-interaction between the two state variables, the quadratic terms increase local nonlinearity, and the folding operator enhances state redistribution. These components jointly determine the dynamical behavior of the proposed map.

To further evaluate the performance of the proposed 2D-FSLM chaotic system, it is compared with several representative two-dimensional chaotic systems, including 2D-LCCCM [39], 2D-SCLM [40], and 2D-SLEM [29]. The specific mathematical definitions of these systems are summarized in Table 1 for clarity.

#### 2.2.2. Bifurcation Analysis

**Figure 2:**
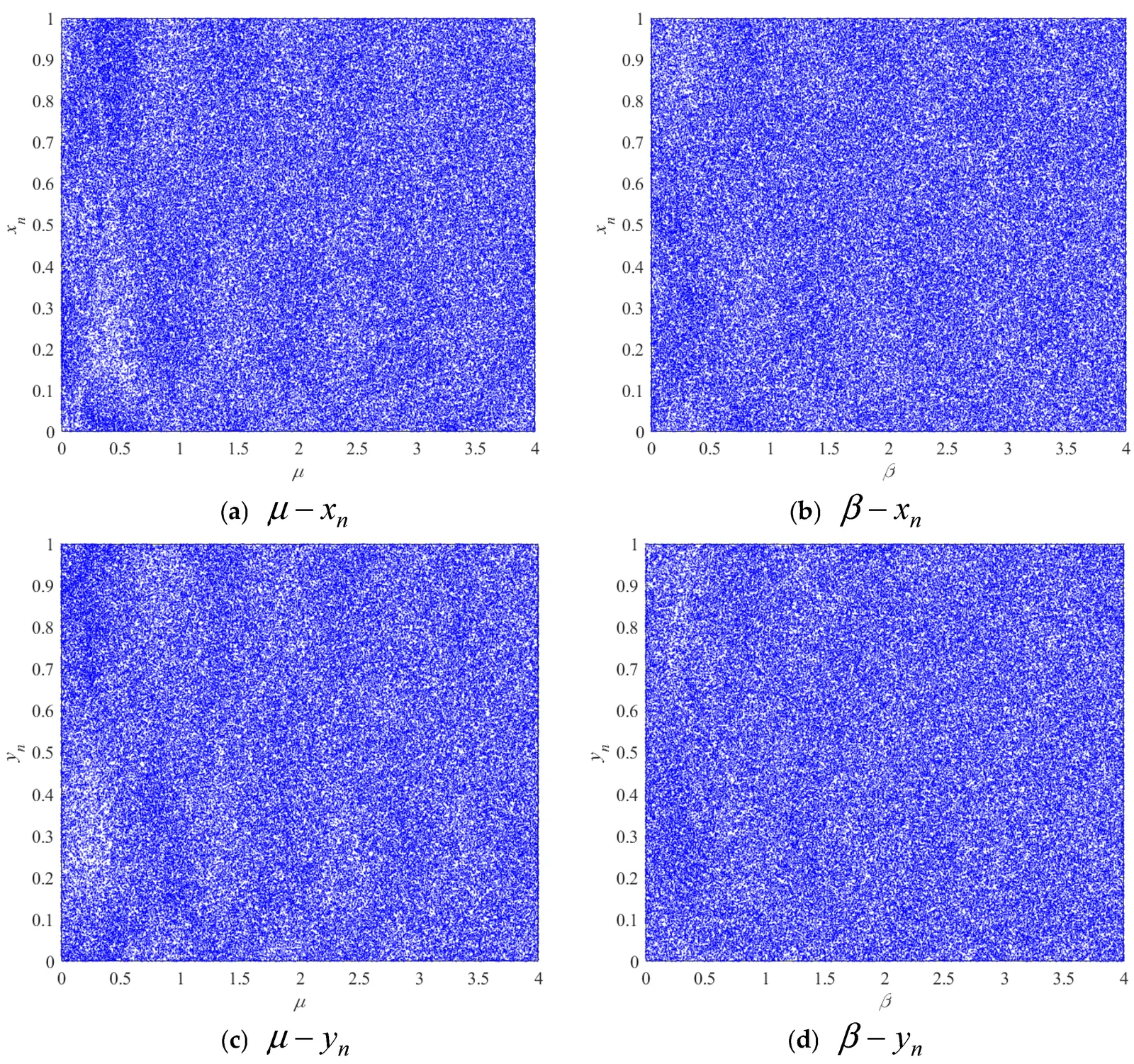
Bifurcation diagrams of the proposed 2D-FSLM.

Bifurcation analysis is an effective tool for examining the variation of system dynamics with respect to control parameters. A broad and dense bifurcation distribution usually indicates richer dynamical behavior and better parameter flexibility. Figure 2 shows the bifurcation diagrams of the proposed 2D-FSLM with respect to the selected control parameter. Specifically, in Figure 2a,c, the parameter β is fixed at 0.43 while μ varies within the prescribed range; in Figure 2b,d, the parameter μ is fixed at 0.97 while β varies. As shown in Figure 2, the iterated points remain densely distributed over most of the parameter interval and cover nearly the entire state range (0,1), indicating that the proposed map maintains complex dynamical behavior over a relatively wide parameter region.

#### 2.2.3. Trajectory Analysis

**Figure 3:**
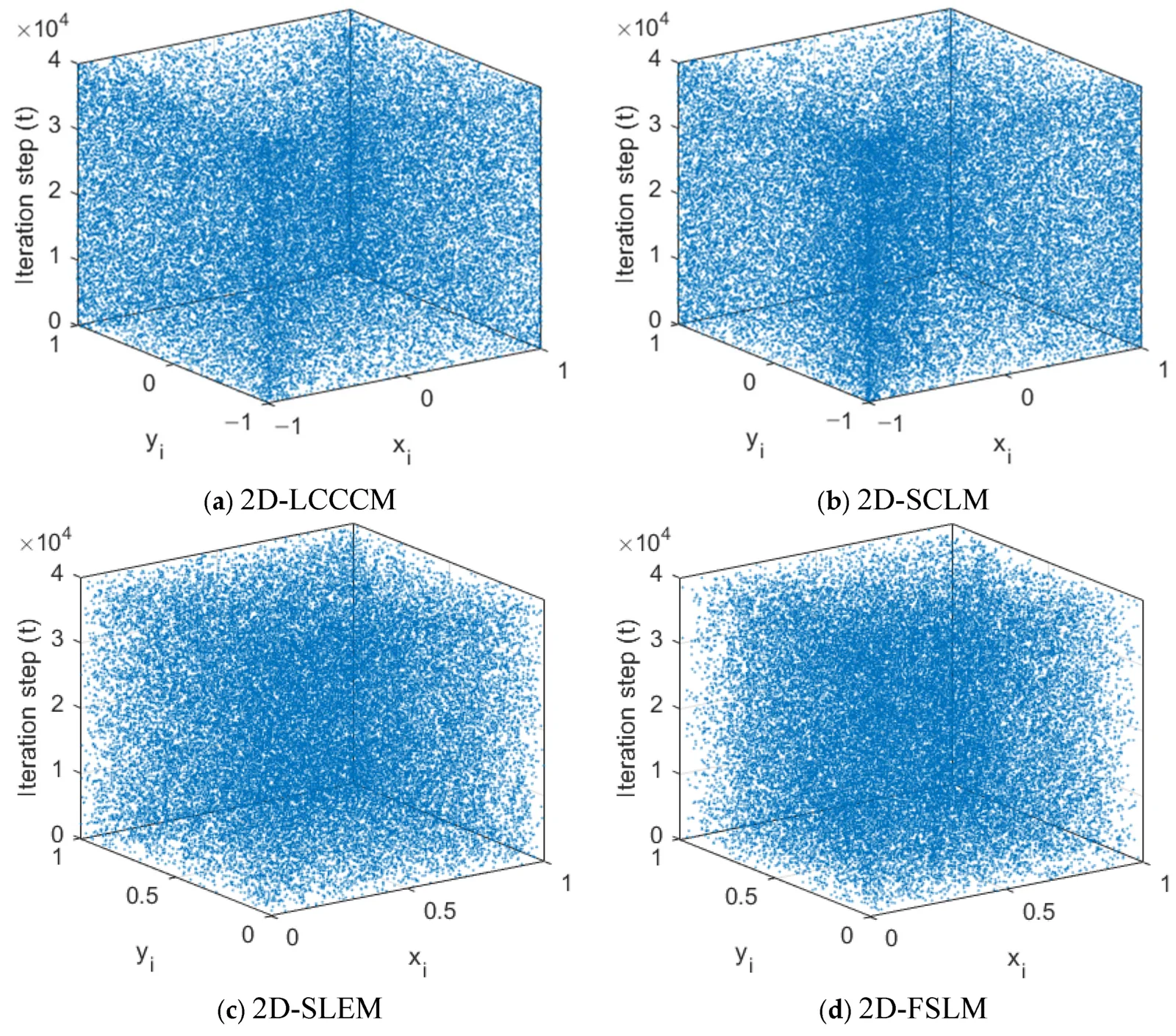
Trajectory diagrams of the comparison maps.

The trajectory diagram can further reveal the continuous evolution path of system states in the phase space. Compared with a scatter-type phase portrait, the trajectory diagram reflects the motion pattern of the orbit during iteration and is useful for observing whether the system exhibits excessive regularity, local trapping, or insufficient spreading. Figure 3a, Figure 3b, and Figure 3c correspond to the trajectory distributions of 2D-LCCCM, 2D-SCLM, and 2D-SLEM, respectively, while Figure 3d presents the trajectory diagram of the proposed 2D-FSLM. It can be observed that the comparison maps in Figure 3a,b are able to generate chaotic trajectories with a certain degree of state-space coverage. However, their trajectory distributions still show relatively distinguishable density variations or uneven spreading in some regions, indicating that the orbit evolution may have certain local concentration characteristics.

In contrast, the chaotic maps in Figure 3c,d exhibit a more complex and continuously varying trajectory distribution. Therefore, the trajectory analysis visually demonstrates that the proposed 2D-FSLM has favorable dynamic characteristics compared with the existing two-dimensional chaotic maps, which is beneficial for generating pseudo-random sequences in image encryption.

#### 2.2.4. Lyapunov Exponent Analysis

**Figure 4:**
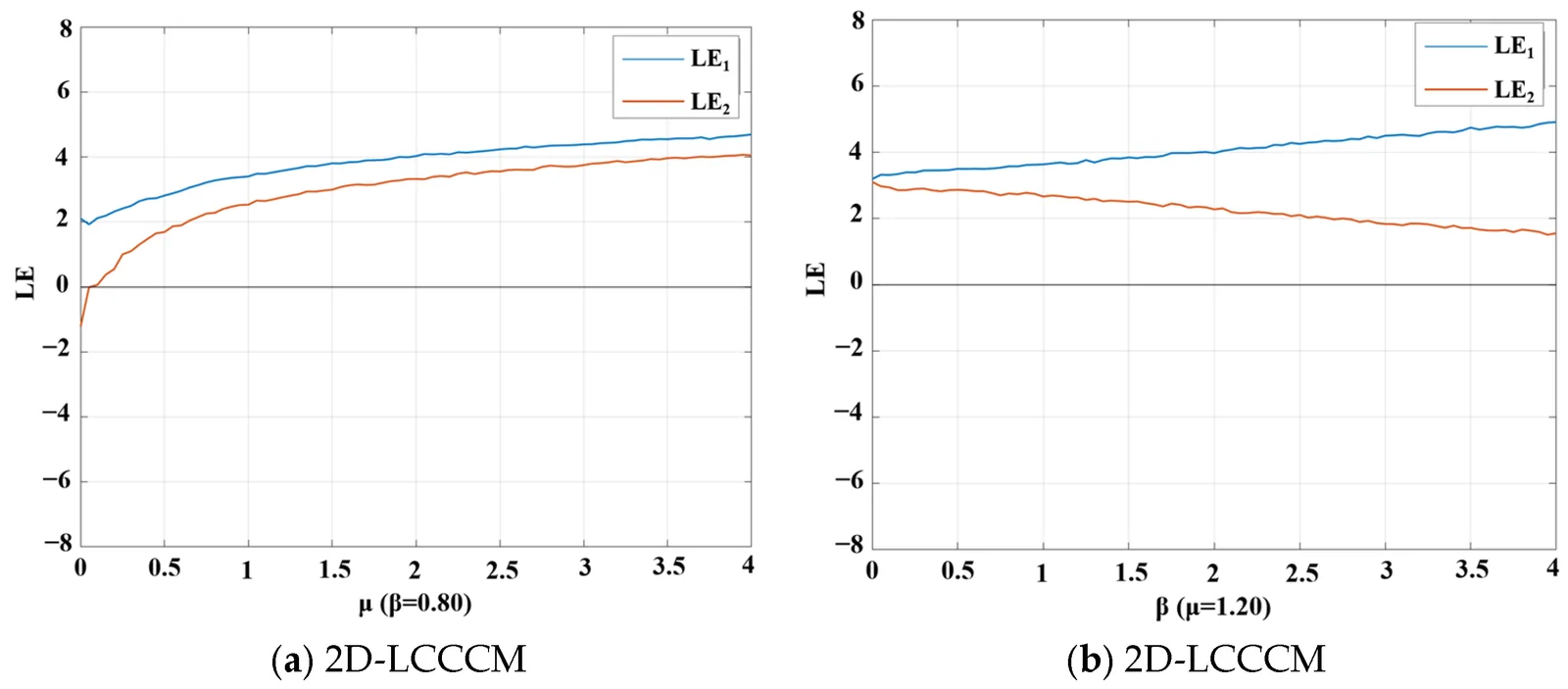

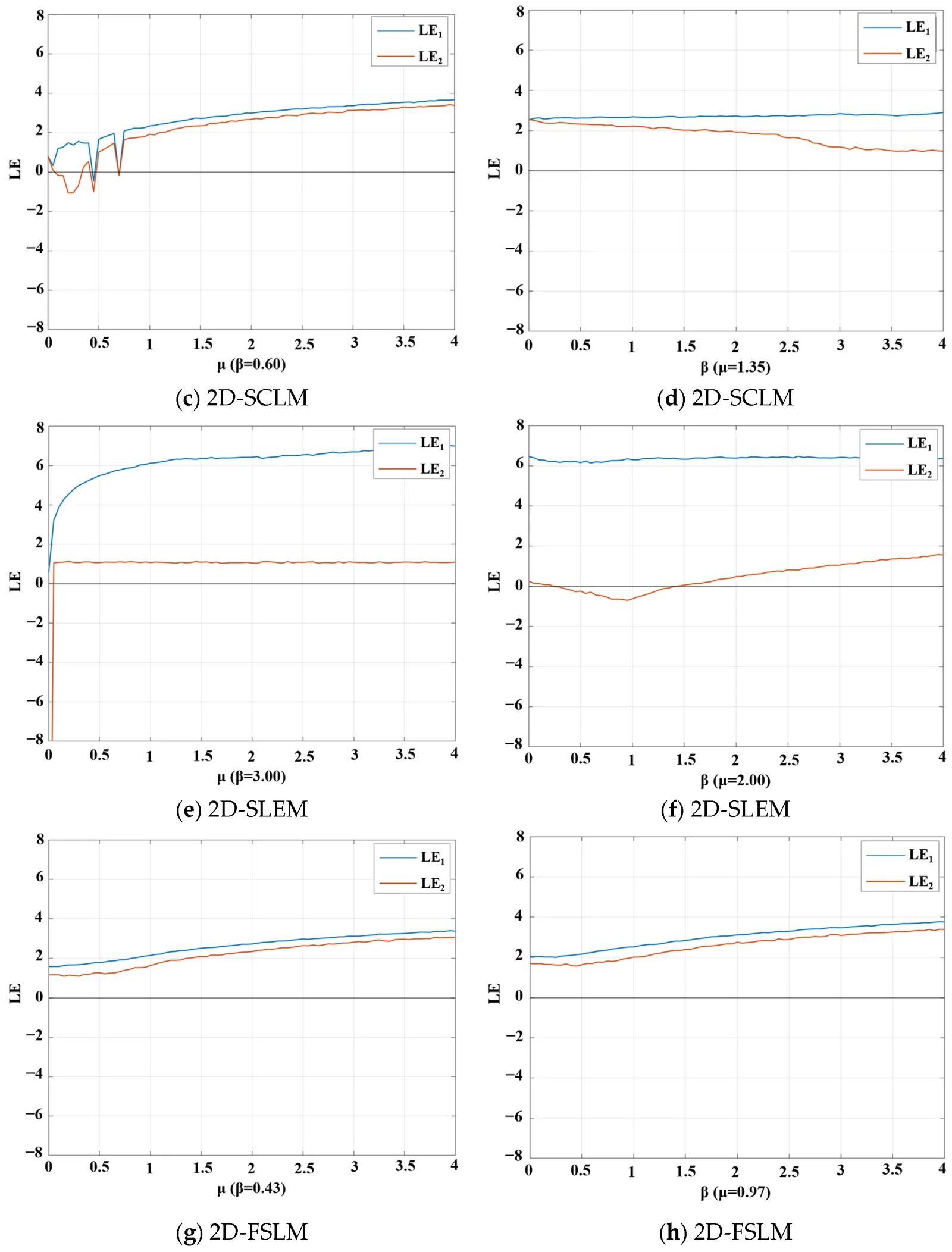
Lyapunov exponent curves of four 2D chaotic maps.

The Lyapunov exponent (*LE*) is used to quantify the divergence of nearby trajectories. Let the proposed system be written in vector form as

(4) $$X_{n+1}=F(X_{n}),X_{n}=\left[\begin{array}{c} x_{n}\\ y_{n} \end{array}\right],$$

where F(•) denotes the nonlinear state-transition mapping defined by Equations (1) and (2). Then the Jacobian matrix at the *n*-th iteration is

(5) $$J_{n}=\left[\begin{array}{cc} \frac{\partial f}{\partial x_{n}} & \frac{\partial f}{\partial y_{n}}\\ \frac{\partial g}{\partial x_{n}} & \frac{\partial g}{\partial y_{n}} \end{array}\right],$$

where f and g denote the two update functions of the map. The Lyapunov exponents are estimated by

(6) $$\lambda_{i}=\underset{N\to \infty }{\lim}\frac{1}{N}\ln\left|\eta_{i}\right|,i=1,2,$$

where $\eta_{i}$ denotes the *i*-th growth factor obtained from the orthogonal decomposition of the Jacobian product. A positive largest Lyapunov exponent indicates exponential divergence of nearby trajectories and therefore chaotic behavior. For a two-dimensional map, two positive Lyapunov exponents indicate divergence in two independent directions and stronger dynamical complexity, whereas a negative exponent indicates contraction in the corresponding direction. Therefore, as shown in Figure 4g,h, Lyapunov exponents that remain positive over a broad parameter range indicate robust chaotic behavior. In computation, the QR-decomposition method is adopted. The two calculated Lyapunov exponents are arranged in descending order as *LE*_1_ ≥ *LE*_2_, where *LE*_1_ is the largest Lyapunov exponent, and *LE*_2_ is the second Lyapunov exponent. For comparison, the Lyapunov exponent curves of 2D-LCCCM, 2D-SCLM, 2D-SLEM, and the proposed 2D-FSLM are shown in Figure 4a–h. In each case, the left subfigure presents the μ-scan, in which μ varies from 0 to 4 while β is fixed, whereas the right subfigure presents the β-scan, in which β varies from 0 to 4 while μ is fixed. As shown in Figure 4a–h, the four maps exhibit different Lyapunov exponent distributions over the scanned parameter intervals. For 2D-LCCCM and 2D-SCLM, both exponents remain positive in most of the considered range, indicating sustained chaotic behavior under the selected parameter settings. For 2D-SLEM, *LE*_1_ remains positive throughout the scan, whereas *LE*_2_ becomes negative in part of the β-scan, suggesting that the corresponding dynamics are less stable in that region. In contrast, the proposed 2D-FSLM maintains two positive Lyapunov exponents in both the μ-scan and the β-scan, and the corresponding curves vary smoothly without obvious degradation. This indicates that the proposed map preserves a relatively stable dual-positive Lyapunov structure over a broad parameter range.

#### 2.2.5. Sample Entropy Analysis

Sample entropy is used to quantify the irregularity and complexity of a sequence. For encryption-oriented chaotic outputs, a larger sample entropy generally indicates lower regularity and stronger sequence complexity. Given a sequence ${\left\{u(i)\right\}}_{i=1}^{N}$, the sample entropy is defined as

(7) $$SampEn(m,r,N)=-\ln(\frac{A^{m}(r)}{B^{m}(r)}),$$

where m is the embedding dimension, r is the tolerance, $B^{m}(r)$ denotes the average matching probability for embedding dimension m, and $A^{m}(r)$ denotes the corresponding average matching probability for dimension m+1.

**Figure 5:**
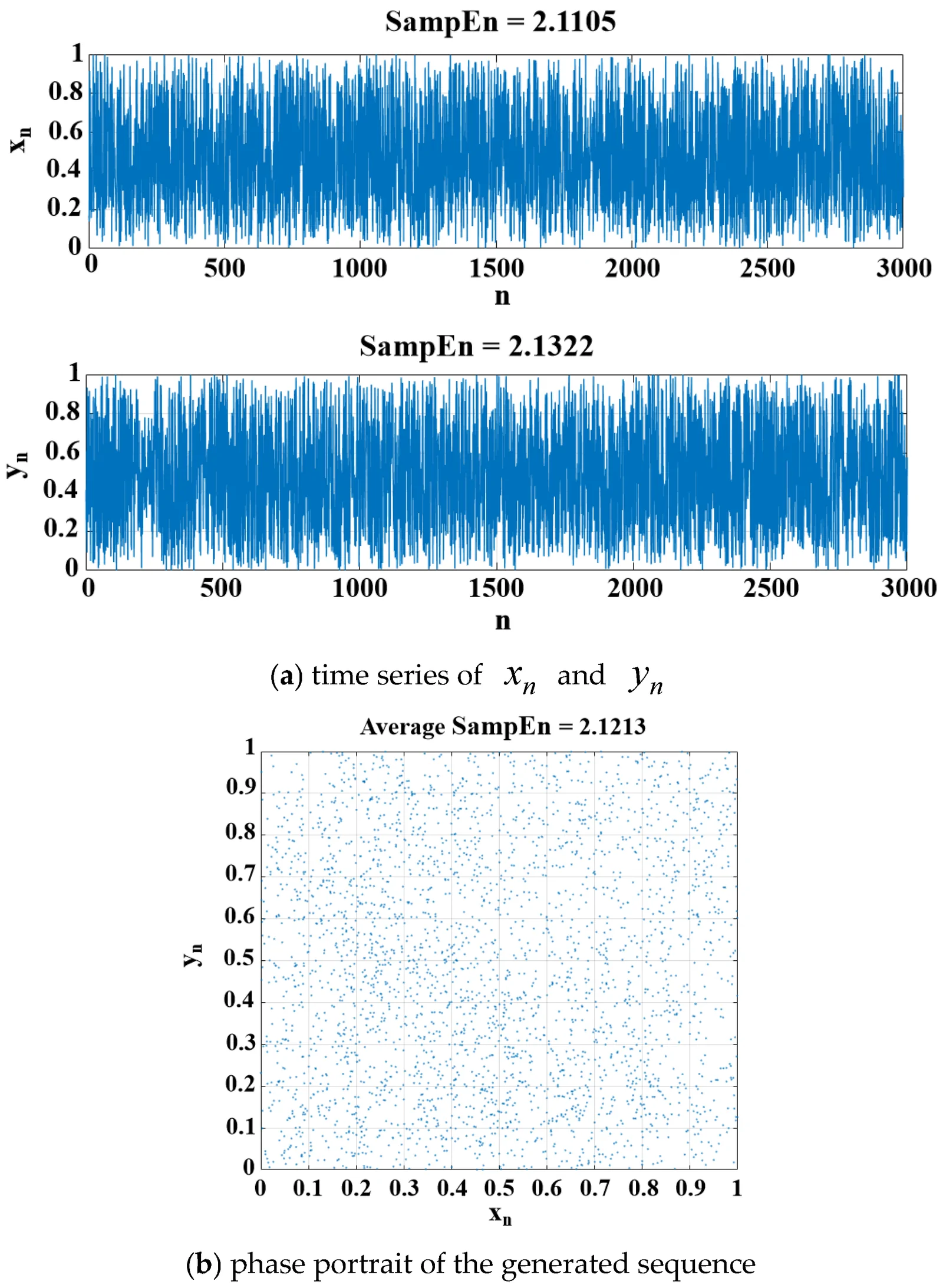
Sample entropy analysis of the proposed 2D-FSLM.

Based on the Lyapunov exponent results in Figure 4g,h, μ = 0.97 and β = 0.43 are selected as a representative operating point within a stable region where both Lyapunov exponents remain positive. Under this parameter setting, the sample entropy values of $x_{n}$ and $y_{n}$ are calculated as 2.1105 and 2.1322, respectively, with an average value of 2.1213. As shown in Figure 5a, both time series exhibit strong fluctuations without visible periodic structures, which is consistent with the obtained sample entropy values. In addition, Figure 5b shows that the iterated points are scattered over most regions of the state space (0,1) × (0,1) without obvious aggregation, further indicating that the generated sequences possess good irregularity and state-space dispersion. Therefore, the sample entropy results support the ability of the proposed 2D-FSLM to generate sequences with relatively high complexity for encryption-oriented applications.

#### 2.2.6. Sensitivity to Control Parameters and Initial Values

Sensitivity to control parameters and initial values is a fundamental property of chaotic systems. For encryption-oriented applications, a tiny perturbation in the system parameters or initial state should lead to a significant divergence in the generated sequences. This property is closely related to key sensitivity and is important for enhancing resistance against parameter estimation and reconstruction attacks.

**Figure 6:**
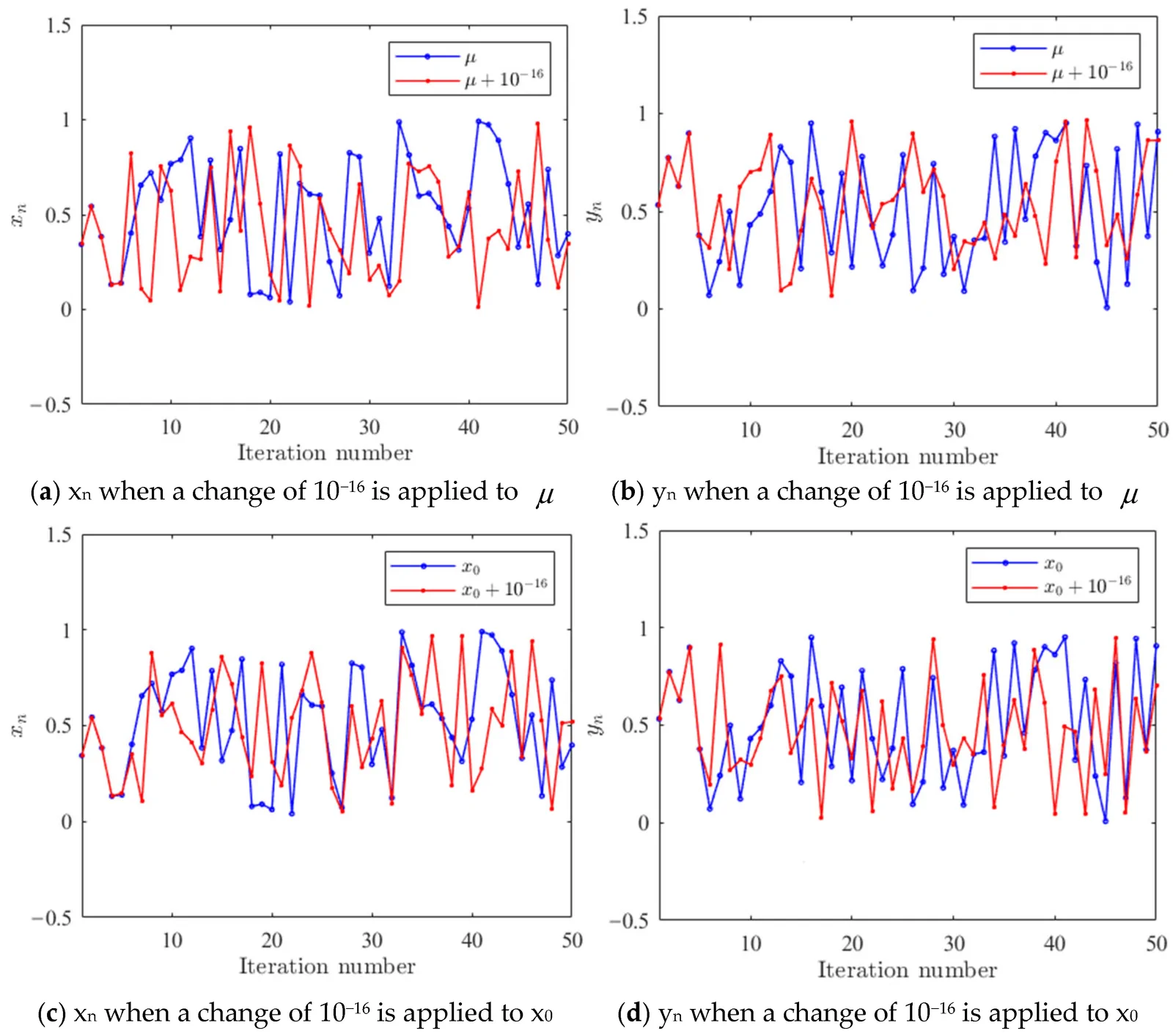
The chaotic sequences with different control parameters and initial values.

Preliminary numerical tests identified μ = 0.01 and β = 47.3 as a representative parameter setting for evaluating the sensitivity of the proposed 2D-FSLM. Under a tiny perturbation, the original and perturbed sequences exhibit a clearly observable divergence after approximately six iterations, thereby allowing the sensitivity of the proposed map to be demonstrated more clearly. Figure 6a,b show the sequences of $x_{n}$ and $y_{n}$ when a perturbation of 10^−16^ is applied to μ, whereas Figure 6c,d show the corresponding sequences when a perturbation of 10^−16^ is applied to $x_{0}$. As can be observed, although the perturbations are extremely small, the original and perturbed sequences become noticeably different. From the viewpoint of image encryption, such sensitivity is desirable because an extremely small deviation in the secret key or initial condition can lead to significantly different chaotic outputs, thereby improving the key sensitivity and security of the encryption scheme.

#### 2.2.7. 0-1 Test for Chaos

The 0-1 test [13] is a time-series-based method for distinguishing chaotic dynamics from regular or periodic dynamics without phase-space reconstruction or explicit calculation of Lyapunov exponents. It transforms a scalar sequence into a trajectory in the p−q plane. A bounded trajectory indicates regular or periodic dynamics, whereas an unbounded Brownian-like trajectory indicates chaotic dynamics. Given a scalar sequence φ(j), the transformed translation variables are defined as

(8) $$\left\{\begin{array}{c} p_{c}(n)=\sum_{j=1}^{n}\varphi (j)\cos(jc),\\ q_{c}(n)=\sum_{j=1}^{n}\varphi (j)\sin(jc), \end{array}\right.$$

where c∈(0,π) is an auxiliary constant. For each auxiliary parameter c, the mean square displacement is calculated as

(9) $$M_{c}(t)=\frac{1}{N-t}\sum_{j=1}^{N-t}\left({\left[p(j+t)-p(j)\right]}^{2}+{\left[q(j+t)-q(j)\right]}^{2}\right).$$

The corresponding statistic $K_{c}$ is then defined as the Pearson correlation coefficient between the displacement step t and $M_{c}(t)$. When $M_{c}(t)$ remains bounded, $K_{c}$ is close to 0, indicating regular or periodic dynamics. When $M_{c}(t)$ grows approximately linearly with t, $K_{c}$ is close to 1, indicating chaotic dynamics.

**Figure 7:**
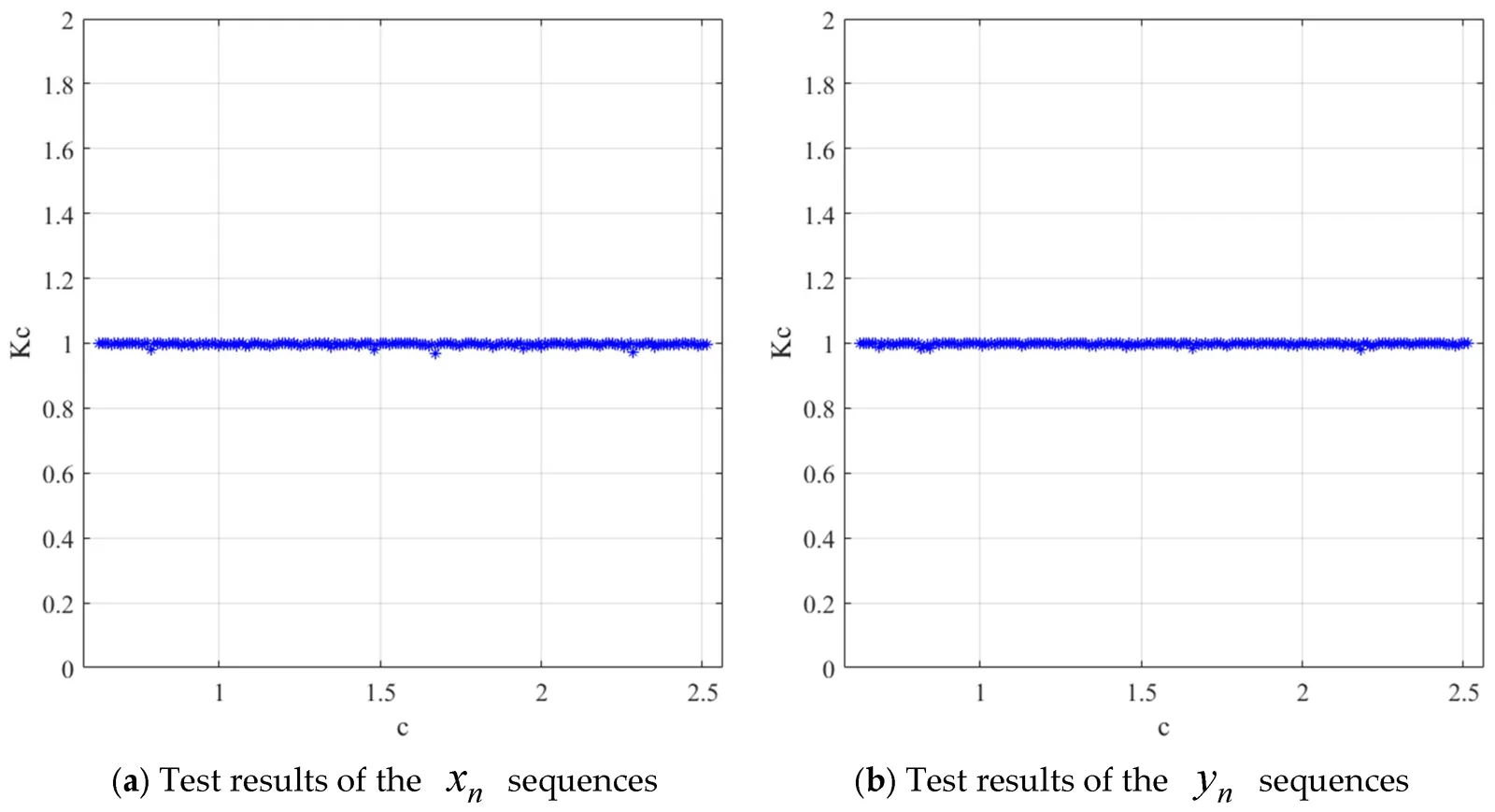
0-1 chaos test.

The 0-1 test is applied to the normalized $x_{n}$ and $y_{n}$ sequences. As shown in Figure 7a,b, $K_{c}$ remains close to 1 over the sampled values of c for both sequences, confirming the chaotic behavior of the proposed 2D-FSL.M.

#### 2.2.8. NIST Randomness Analysis

To further evaluate the statistical randomness of the binary sequences generated by the proposed 2D-FSLM, the official NIST SP 800-22 Rev 1a test suite was applied to the X sequence and the Y sequence. Table 2 summarizes the results in terms of the 15 standard NIST test families. These results indicate that the X and Y binary sequences generated by the proposed 2D-FSLM possess good statistical randomness and are suitable for encryption-oriented applications.

Overall, the proposed 2D-FSLM can be evaluated from multiple perspectives, including qualitative trajectory behavior, parameter-dependent dynamics, sensitivity characteristics, chaos identification, and statistical randomness. These analyses provide a solid basis for employing the proposed map in the subsequent image permutation and diffusion stages.

## 3. Proposed Image Encryption Algorithm

### 3.1. Overall Framework

Let the plaintext image be denoted by

(10) $$P\in {\left\{0,1,\ldots ,255\right\}}^{m\times n\times ch},$$

where *m* and *n* denote the number of rows and columns of the image, respectively, and *ch* denotes the number of channels, with *ch* = 1 for a grayscale image and *ch* = 3 for an RGB image. The corresponding ciphertext image is denoted by *C*. The proposed encryption algorithm is driven by the 2D-FSLM and consists of plaintext-related session-key generation, concentric-ring permutation, and enhanced hybrid diffusion.

For convenience, the *k*-th channel of the plaintext image is vectorized as

(11) $$p^{\left(k\right)}={\left\{p_{i}^{\left(k\right)}\right\}}_{i=1}^{L},L=m\times n,k=1,\ldots ,ch.$$

Similarly, the ciphertext sequence of the *k*-th channel is denoted by

(12) $$c^{\left(k\right)}={\left\{c_{i}^{\left(k\right)}\right\}}_{i=1}^{L}.$$

**Figure 8:**
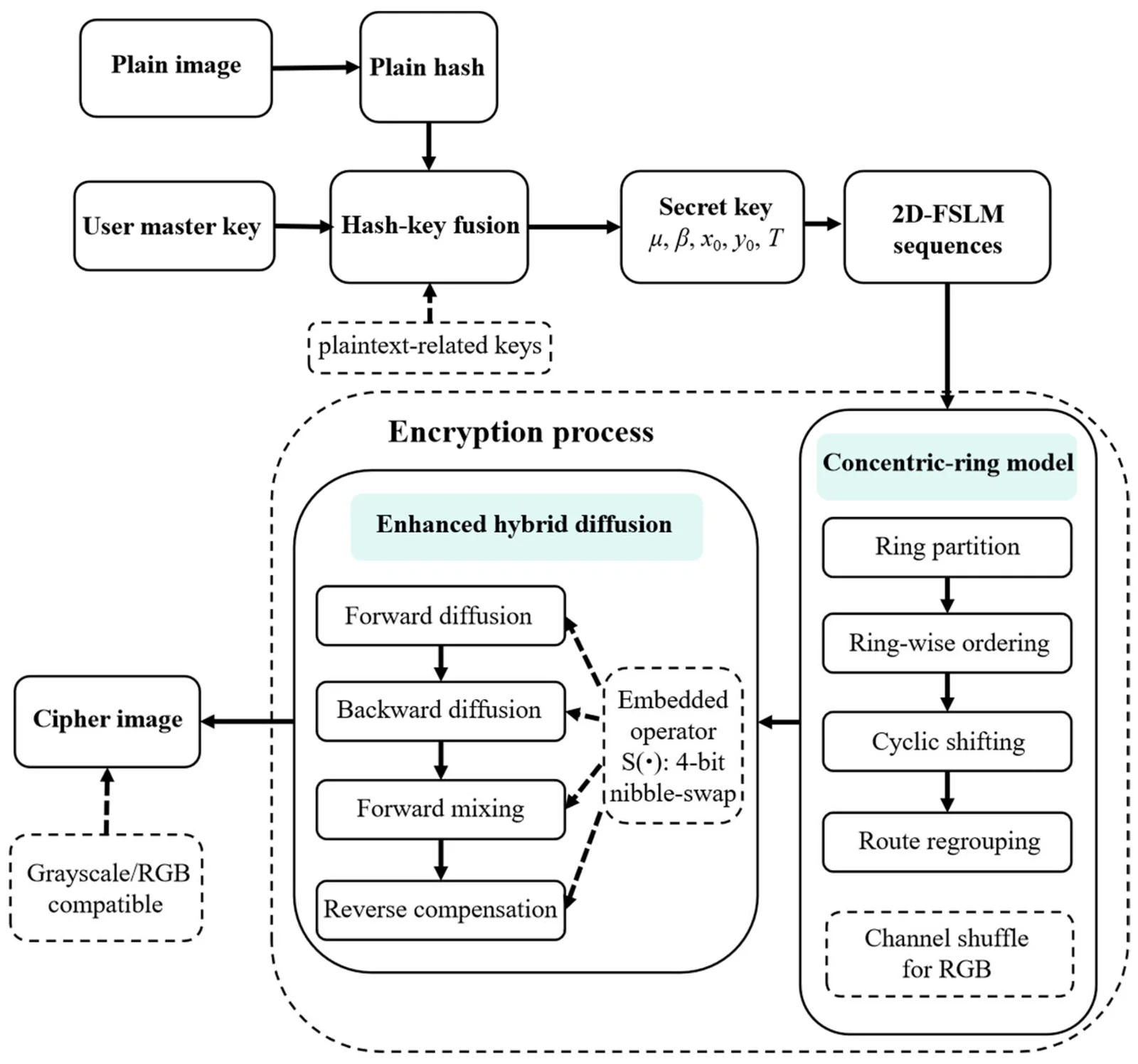
Overall framework of the proposed image encryption algorithm.

As shown in Figure 8, the proposed image encryption algorithm consists of plaintext-related session-key generation, concentric-ring model, and enhanced hybrid diffusion. First, the user key and the SHA-256 hash of the plaintext image are jointly used to derive the session parameters of the proposed 2D-FSLM. Then, the generated chaotic sequences are used to control pixel permutation and value diffusion. For RGB images, the spatial permutation is jointly applied to aligned RGB pixel vectors, and a channel shuffle is further introduced at each pixel position. After that, the diffusion stage is carried out channel by channel. In this way, the proposed scheme provides both spatial scrambling and inter-channel confusion. It should be noted that the proposed permutation strategy only draws on the multi-ring circulation and entrance-exit routing mechanism of the overpass. In the actual algorithm, three rings and three chaos-controlled exits are adopted.

### 3.2. Key Generation

To make the encryption process plaintext-dependent, the session parameters of the 2D-FSLM are generated jointly from the user key and the plaintext image. The detailed process consists of the following steps.

Step 1 plaintext hash generation: Input the plaintext image and convert it into a byte stream. Then, the SHA-256 hash function is applied to the byte stream to obtain a 64-digit hexadecimal plaintext hash.

Step 2 mixed hash generation and normalization: Concatenate the user master key with the plaintext hash, and apply SHA-256 again to generate a mixed hash string. The resulting 64-digit hexadecimal string is divided into 32 bytes. Let $m_{i}$ denote the *i*-th byte of the mixed hash, where $m_{i}$ belongs to {0, 1, …, 255}. Each byte is normalized as

(13) $$h_{i}=\frac{m_{i}+0.5}{256},i=1,2,\ldots ,32.$$

Step 3 session parameter derivation: According to the normalized hash values, the control parameters and initial values of the 2D-FSLM are generated as

(14) $$\left\{\begin{array}{l} \mu =0.90+0.14\times (\sum_{i=1}^{7}h_{i}/7),\\ \beta =0.35+0.16\times (\sum_{i=8}^{14}h_{i}/7),\\ x_{0}=\mathrm{mod}(\sum_{i=15}^{17}h_{i}/3+0.3\times (\sum_{i=18}^{21}h_{i}/4),1),\\ y_{0}=\mathrm{mod}(\sum_{i=22}^{25}h_{i}/4+0.7\times (\sum_{i=26}^{28}h_{i}/3),1),\\ T=1500+\mathrm{mod}(\left\lfloor 10^{10}\times \sum_{i=29}^{32}h_{i}/4\right\rfloor ,1500). \end{array}\right.$$

Step 4 chaotic sequence generation: After discarding the first T transient states, the 2D-FSLM is further iterated to generate two chaotic output sequences ${\left\{x_{i}\right\}}_{i=1}^{2L}$ and ${\left\{y_{i}\right\}}_{i=1}^{2L+3}$. The first part of the chaotic outputs is used for permutation,

(15) $$x^{\left(p\right)}={\left\{x_{i}\right\}}_{i=1}^{L},\;\;\;y^{\left(p\right)}={\left\{y_{i}\right\}}_{i=1}^{L+3},$$

and the remaining part is used for diffusion,

(16) $$x^{\left(d\right)}={\left\{x_{i}\right\}}_{i=L+1}^{2L},\;\;\;y^{\left(d\right)}={\left\{y_{i}\right\}}_{i=L+4}^{2L+3}.$$

Because the session parameters depend on both the user key and the plaintext image, different plaintext images lead to different effective chaotic trajectories even when the same user key is reused. This property improves key sensitivity and strengthens the plaintext dependence of the scheme.

### 3.3. Concentric-Ring Model

To disrupt the spatial correlation of neighboring pixels, a concentric-ring permutation strategy is employed. The detailed process is as follows.

Step 1 image center and polar-coordinate calculation: For an image of size m×n, the image center is defined as

(17) $$(x_{c},y_{c})=(\frac{m+1}{2},\frac{n+1}{2}).$$

For a pixel located at (u,v), its radial distance and polar angle are calculated by

(18) $$\left\{\begin{array}{c} r(u,v)=\sqrt{{\left(u-x_{c}\right)}^{2}+{\left(v-y_{c}\right)}^{2}},\\ \theta (u,v)=\arctan2(u-x_{c},v-y_{c}). \end{array}\right.$$

Step 2 concentric-ring partition: Let $r_{\max}$ denote the maximum radial distance in the image plane. Two thresholds are set as

(19) $$r_{1}=\frac{r_{\max}}{3},r_{2}=\frac{2r_{\max}}{3}.$$

Accordingly, all pixels are divided into three concentric regions,

(20) $$\left\{\begin{array}{l} \Omega_{1}=\{(u,v)|r(u,v)\leq r_{1}\},\\ \Omega_{2}=\{(u,v)|r_{1}<r(u,v)\leq r_{2}\},\\ \Omega_{3}=\{(u,v)|r(u,v)>r_{2}\}, \end{array}\right.$$

Step 3 ring-wise ordering and cyclic shifting: Within each ring, the pixels are ordered lexicographically according to (θ,r,l), where l=u+(v−1)m is the linear index obtained by column-wise vectorization of the original image and uniquely identifies the pixel at row u and column v. Let $n_{j}=\left|\Omega_{j}\right|$ for j=1,2,3. The cyclic shift steps of the three rings are generated from the first three elements of $y^{\left(p\right)}$ as

(21) $$\left\{\begin{array}{c} s_{1}=1+\mathrm{mod}(\left\lfloor 10^{14}\times y_{1}^{\left(p\right)}\right\rfloor ,n_{1}),\\ s_{2}=1+\mathrm{mod}(\left\lfloor 10^{14}\times y_{2}^{\left(p\right)}\right\rfloor ,n_{2}),\\ s_{3}=1+\mathrm{mod}(\left\lfloor 10^{14}\times y_{3}^{\left(p\right)}\right\rfloor ,n_{3}). \end{array}\right.$$

In the implementation, the inner, middle, and outer rings are cyclically shifted by +*S*_1_, −*S*_2_, and +*S*_3_, respectively.

Step 4 chaos-controlled input routing: After ring-wise shifting, route-controlled regrouping is introduced. The input route label of each pixel is determined by

(22) $$a_{i}=\left\{\begin{array}{l} \begin{array}{ll} 1 & x_{i}^{\left(p\right)}<\frac{1}{3} \end{array}\\ \begin{array}{ll} 2 & \frac{1}{3}\leq x_{i}^{\left(p\right)}<\frac{2}{3} \end{array}\\ \begin{array}{ll} 3 & x_{i}^{\left(p\right)}\geq \frac{2}{3} \end{array} \end{array}\right.,\;\;i=1,2,\ldots ,L$$

Thus, all pixels are divided into three input groups, whose sizes are denoted by $n_{1}$, $n_{2}$ and $n_{3}$, respectively.

Step 5 balanced output route assignment: To ensure a bijective mapping and reduce the need to store intermediate routing information during decryption, the output route labels are assigned in a balanced manner. Specifically, the positions are first sorted in ascending order of ${\left\{y_{i}\right\}}_{i=4}^{L+3}$. Then, the first $n_{1}$ positions are assigned to route 1, the next $n_{2}$ positions are assigned to route 2, and the remaining $n_{3}$ positions are assigned to route 3.

Step 6 intra-group ordering and route regrouping: Within each group, pixels are further ordered according to the mixed chaotic key

(23) $$\kappa_{i}=\mathrm{mod}(0.75x^{\left(p\right)}+0.25y^{\left(p\right)},1).$$

**Figure 9:**
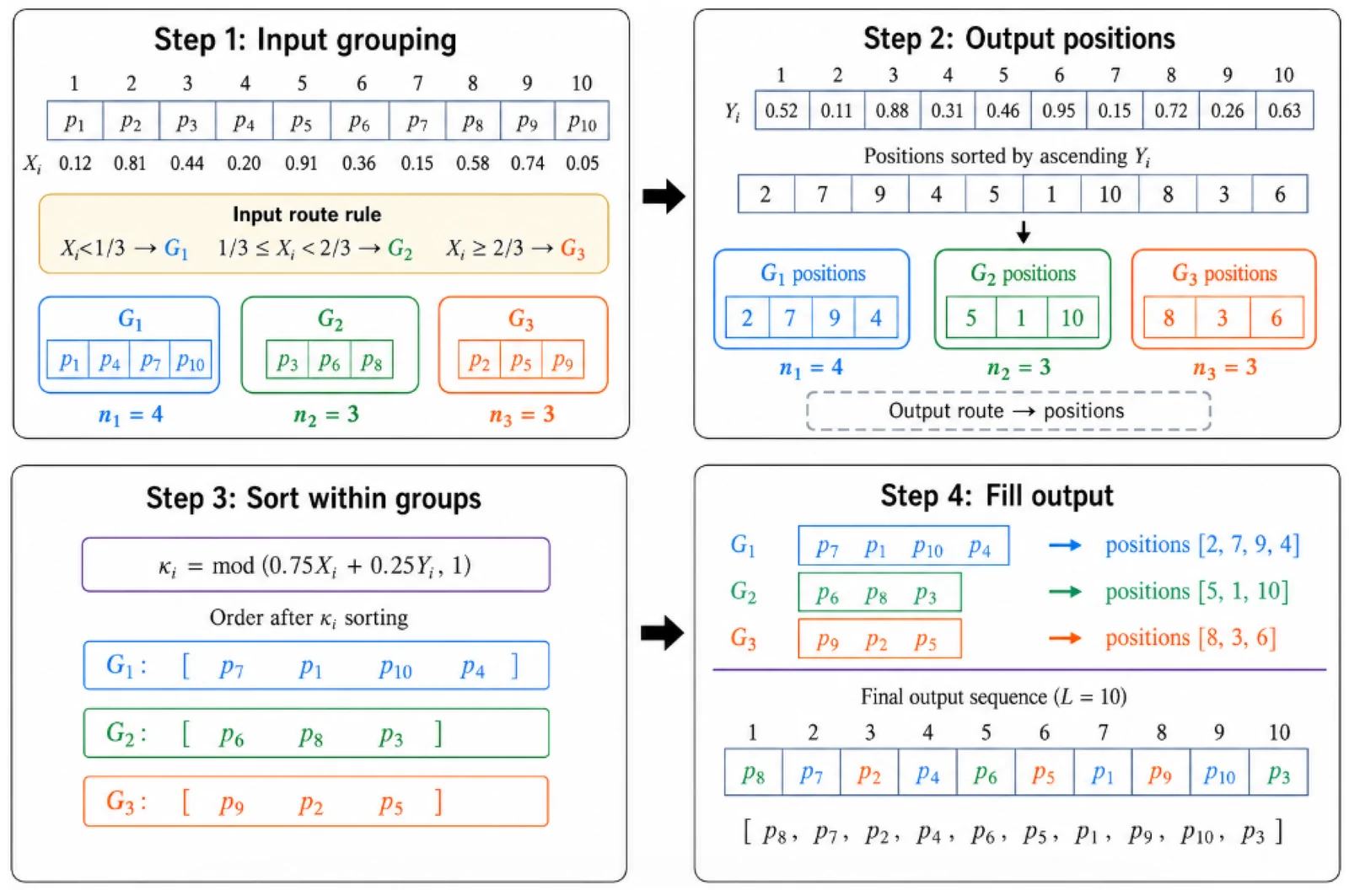
Illustration of the balanced route regrouping strategy.

After group-wise ordering and balanced route regrouping, the final spatially permuted image is obtained. In this way, the proposed concentric-ring permutation not only performs ring-wise scrambling but also introduces cross-region pixel migration. Figure 9 depicts the implementation of the balanced route regrouping strategy.

Step 7 channel permutation for color images: For a grayscale image, the above operation is directly applied to the single-channel pixel sequence. For a color image, the aligned RGB triplet at each spatial position is first moved jointly. Let the RGB vector at position i be

(24) $$\nu_{i}=(R_{i},G_{i},B_{i}).$$

Then, a chaos-controlled channel permutation is introduced. The channel permutation index is defined by

(25) $$\sigma_{i}=1+\mathrm{mod}(\left\lfloor 10^{14}\times z_{i}\right\rfloor ,6),$$

where

(26) $$z_{i}=\mathrm{mod}(0.62x^{\left(p\right)}+0.38y^{\left(p\right)},1).$$

**Figure 10:**
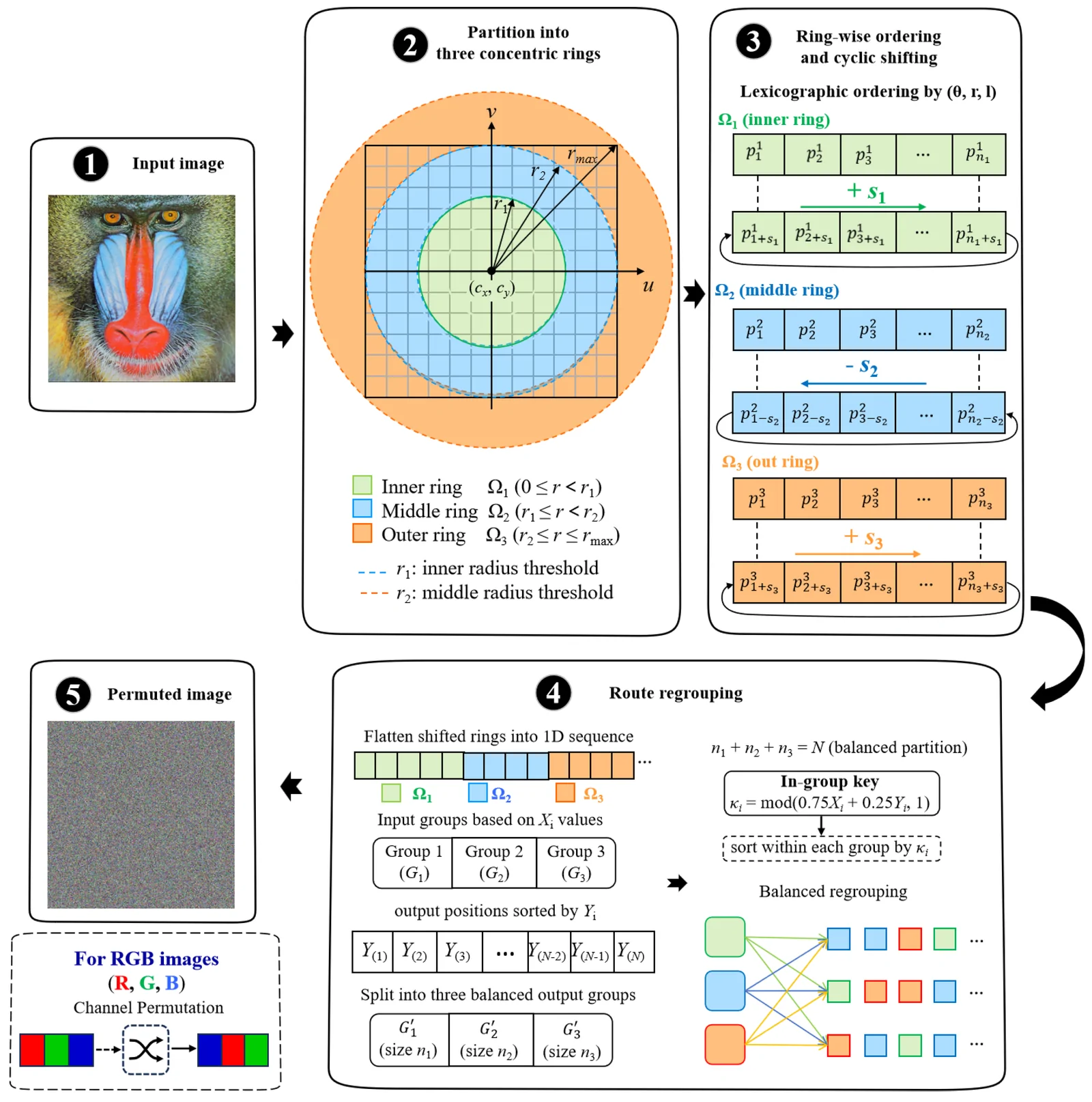
Schematic diagram of the concentric-ring model.

Here, $\sigma_{i}\in \{1,2,\ldots ,6\}$ corresponds to one of the six possible permutations of the RGB components. Therefore, for color images, the permutation stage changes both the spatial positions and the channel arrangement of pixel values. Figure 10 presents a schematic diagram of the proposed concentric-ring model, which enhances spatial scrambling by combining concentric partition, ring-wise processing, and cross-region regrouping.

### 3.4. Diffusion Mask and Seed Generation

After permutation, the second part of the chaotic outputs is used to generate diffusion masks and initial diffusion seeds. These masks and seeds determine the subsequent pixel-value diffusion process.

Step 1 diffusion mask generation: Four integer mask sequences are generated from $x^{\left(d\right)}$ and $y^{\left(d\right)}$ as

(27) $$\left\{\begin{array}{l} K_{i}^{\left(1\right)}=\mathrm{mod}(\left\lfloor x_{i}^{\left(d\right)}\times 10^{14}\right\rfloor ,256),\\ K_{i}^{\left(2\right)}=\mathrm{mod}(\left\lfloor y_{i}^{\left(d\right)}\times 10^{14}\right\rfloor ,256),\\ K_{i}^{\left(3\right)}=\mathrm{mod}(\left\lfloor \mathrm{mod}(x_{i}^{\left(d\right)}+y_{i}^{\left(d\right)},1)\times 10^{14}\right\rfloor ,256),\\ K_{i}^{\left(4\right)}=\mathrm{mod}(\left\lfloor \mathrm{mod}(0.62x_{i}^{\left(d\right)}+0.38y_{i}^{\left(d\right)},1)\times 10^{14}\right\rfloor ,256). \end{array}\right.$$

After reshaping, these four sequences form the mask matrices used in the subsequent diffusion process.

Step 2 plaintext-related statistical term calculation: To further strengthen plaintext dependence, the initial diffusion seeds are generated not only from chaotic sequences but also from the plaintext hash and image statistics. Let $b_{j}$ denote the *j*-th byte of the plaintext SHA-256 hash. Two auxiliary statistical terms are defined as

(28) $$\left\{\begin{array}{l} \tau_{1}=\mathrm{mod}(\sum P+m+n,256),\\ \tau_{2}=\mathrm{mod}(\sum P^{\left(\pi \right)}+2m+3n,256). \end{array}\right.$$

Here, ∑P and $\sum P^{\left(\pi \right)}$ denote the sums of all pixel values in the original and permuted images, respectively. For an RGB image, the summation is performed over all three channels.

Step 3 initial diffusion seed generation: Using the hash bytes and the above statistical terms, the two initial diffusion seeds are calculated as

(29) $$\left\{\begin{array}{c} s_{1}=\mathrm{mod}(b_{1}+b_{11}+\tau_{1},256),\\ s_{2}=\mathrm{mod}(b_{2}+b_{17}+\tau_{2},256). \end{array}\right.$$

Therefore, the initial state of diffusion depends jointly on the plaintext hash, image size, and image statistics.

Step 4 channel-dependent seed update: For RGB images, the four mask sequences $K^{\left(1\right)}\text{–}K^{\left(4\right)}$ are shared by the three channels, while the channel-dependent seeds are updated as

(30) $$\left\{\begin{array}{c} s_{1}^{\left(k\right)}=\mathrm{mod}(s_{1}+31(k-1),256),k=1,2,3,\\ s_{2}^{\left(k\right)}=\mathrm{mod}(s_{2}+47(k-1),256),k=1,2,3. \end{array}\right.$$

In this way, the three channels use the same mask structure but different initialization states, which helps enhance the diversity of channel-wise diffusion.

### 3.5. Enhanced Hybrid Diffusion

To further modify pixel values and enhance the avalanche effect, an enhanced hybrid diffusion mechanism is applied after permutation. This mechanism consists of forward diffusion, backward diffusion, forward mixing, and reverse compensation.

Step 1: Input sequence and nibble-swap operation: Let the permuted sequence of one channel be

(31) $$q=(q_{1},q_{2},\ldots ,q_{L}).$$

To enhance local nonlinearity at low computational cost, a 4-bit nibble-swap operator is introduced:

(32) $$S(z)=16\mathrm{mod}(z,16)+\left\lfloor \frac{z}{16}\right\rfloor ,$$

which exchanges the higher 4 bits and lower 4 bits of the 8-bit integer z. For brevity, ${\left[\cdot \right]}_{256}$ denotes modulo-256 addition, and ⊕ denotes bitwise XOR.

Step 2 forward diffusion: The forward diffusion stage is performed from the first pixel to the last pixel as

(33) $$u_{i}={\left[q_{i}+K_{i}^{\left(1\right)}+u_{i-1}+(K_{i}^{\left(2\right)}⊕u_{i-1})+(K_{i}^{\left(3\right)}⊕S(u_{i-1}))\right]}_{256}⊕(K_{i}^{\left(3\right)}⊕S(u_{i-1})⊕K_{i}^{\left(1\right)}),$$

where $u_{0}=s_{1}.$

Step 3 backward diffusion: The backward diffusion stage is then performed from the last pixel to the first pixel as

(34) $$\begin{array}{l} v_{i}={\left[u_{i}+K_{i}^{\left(4\right)}+v_{i+1}+(K_{i}^{\left(1\right)}⊕v_{i+1})+(K_{i}^{\left(2\right)}⊕S(v_{i+1}))\right]}_{256}⊕({\left[K_{i}^{\left(2\right)}+v_{i+1}+K_{i}^{\left(3\right)}\right]}_{256}\\ ⊕K_{i}^{\left(4\right)}⊕S(v_{i+1})), \end{array}$$

where $v_{L+1}=s_{2}.$

Step 4 forward mixing: Based on the backward diffusion result, a forward mixing stage is further introduced as

(35) $$\omega_{i}={\left[v_{i}+(K_{i}^{\left(4\right)}⊕S(\omega_{i-1}))+\omega_{i-1}+(K_{i}^{\left(1\right)}⊕S(\omega_{i-1}))\right]}_{256},$$

where $\omega_{0}=s_{1}⊕s_{2}.$

Step 5 reverse compensation: Finally, the reverse compensation stage is applied to strengthen the dependency between current and subsequent pixels,

(36) $$c_{i}={\left[\omega_{i}+(K_{i}^{\left(3\right)}⊕S(c_{i+1}))+c_{i+1}+(K_{i}^{\left(2\right)}⊕S(c_{i+1}))\right]}_{256},$$

where $c_{L+1}=s_{1}⊕{\left[s_{2}+91\right]}_{256}.$

For a grayscale image, the above diffusion is executed once. For an RGB image, the same diffusion structure is applied separately to the three channels by using the channel-dependent seeds $s_{1}^{\left(k\right)}$ and $s_{2}^{\left(k\right)}$. Through multi-stage propagation and nonlinear bit-level disturbance, the proposed diffusion mechanism effectively spreads small plaintext changes over the entire ciphertext image.

### 3.6. Decryption Process

For the decryption process, the ciphertext is transmitted over a public channel, and the secret key $\left(\mu ,\beta ,x_{0},y_{0},T\right)$ is sent separately via a secure channel for subsequent decryption by the recipient. Since both the concentric-ring permutation and the enhanced hybrid diffusion are reversible, decryption is performed by applying the corresponding inverse operations in reverse order. The legal recipient first regenerates the same chaotic sequences of the 2D-FSLM from the secret key. Then the same mask sequences and diffusion seeds are reconstructed. After that, inverse reverse compensation, inverse forward mixing, inverse backward diffusion, and inverse forward diffusion are successively carried out to recover the permuted image.

**Figure 11:**
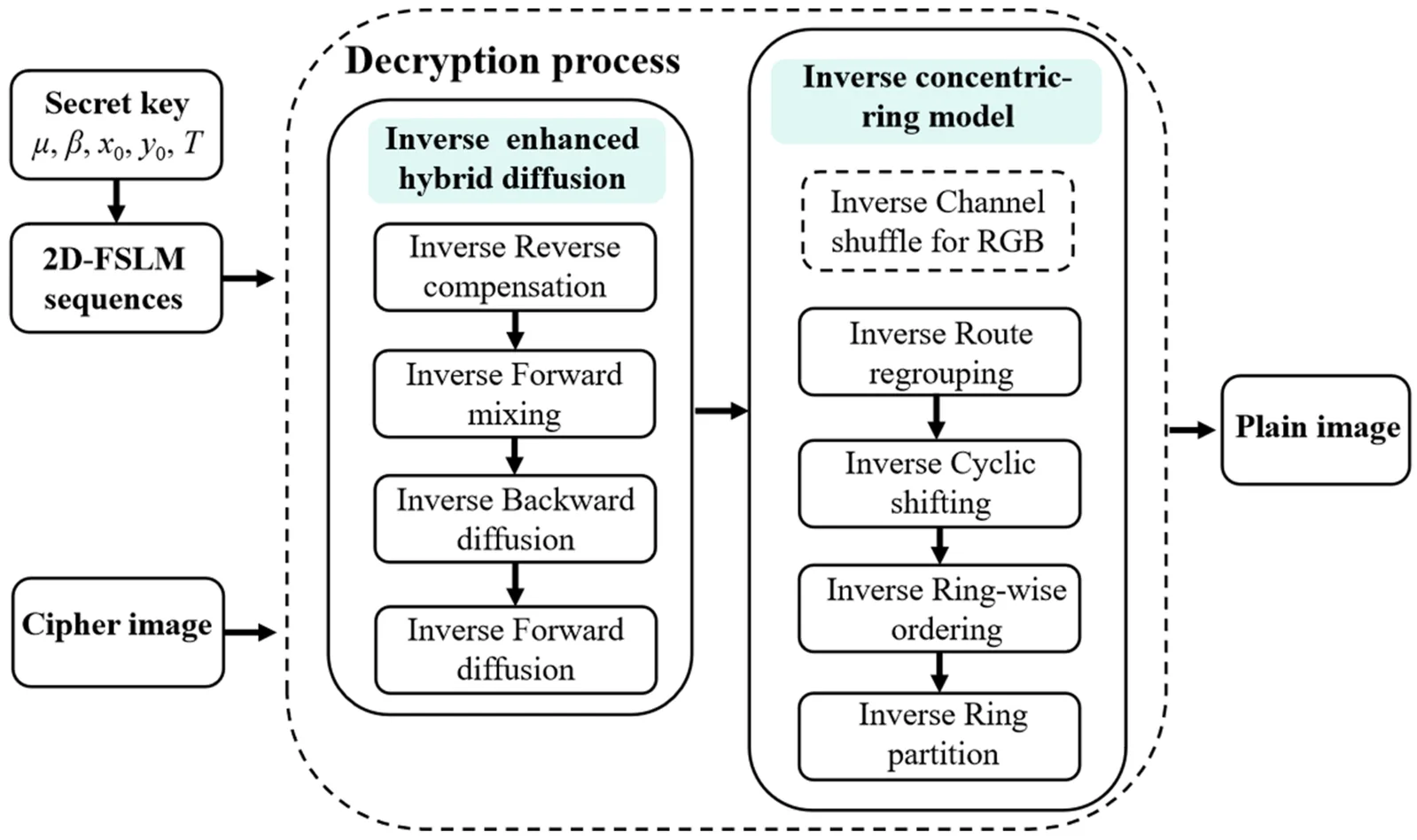
Illustration of decryption process.

For RGB images, after inverse diffusion, the same chaos-controlled channel permutation index $\sigma_{i}$ is regenerated, and the inverse channel shuffle is applied at each pixel position to restore the original RGB arrangement. Then the regrouping process is inversely reconstructed from the same sequences. Finally, ring indices are regenerated from image size m × n and fixed partition rules, and the three rings are circularly shifted in the opposite directions to recover the plaintext image. The decryption process is illustrated in Figure 11.

## 4. Experimental Results and Algorithm Analysis

### 4.1. Experimental Setup and Visual Results

To evaluate the performance of the proposed encryption scheme, all experiments were carried out in the MATLAB R2024b environment. The hardware platform was a 64-bit Windows operating system equipped with a 13th Gen Intel(R) Core(TM) i7-13650HX processor (2.60 GHz) and 32 GB RAM. The test images used in this work were selected from the USC-SIPI image database (https://sipi.usc.edu/database/ (accessed on 8 April 2026)). Representative images were employed to verify the applicability of the proposed method to different image types.

**Figure 12:**
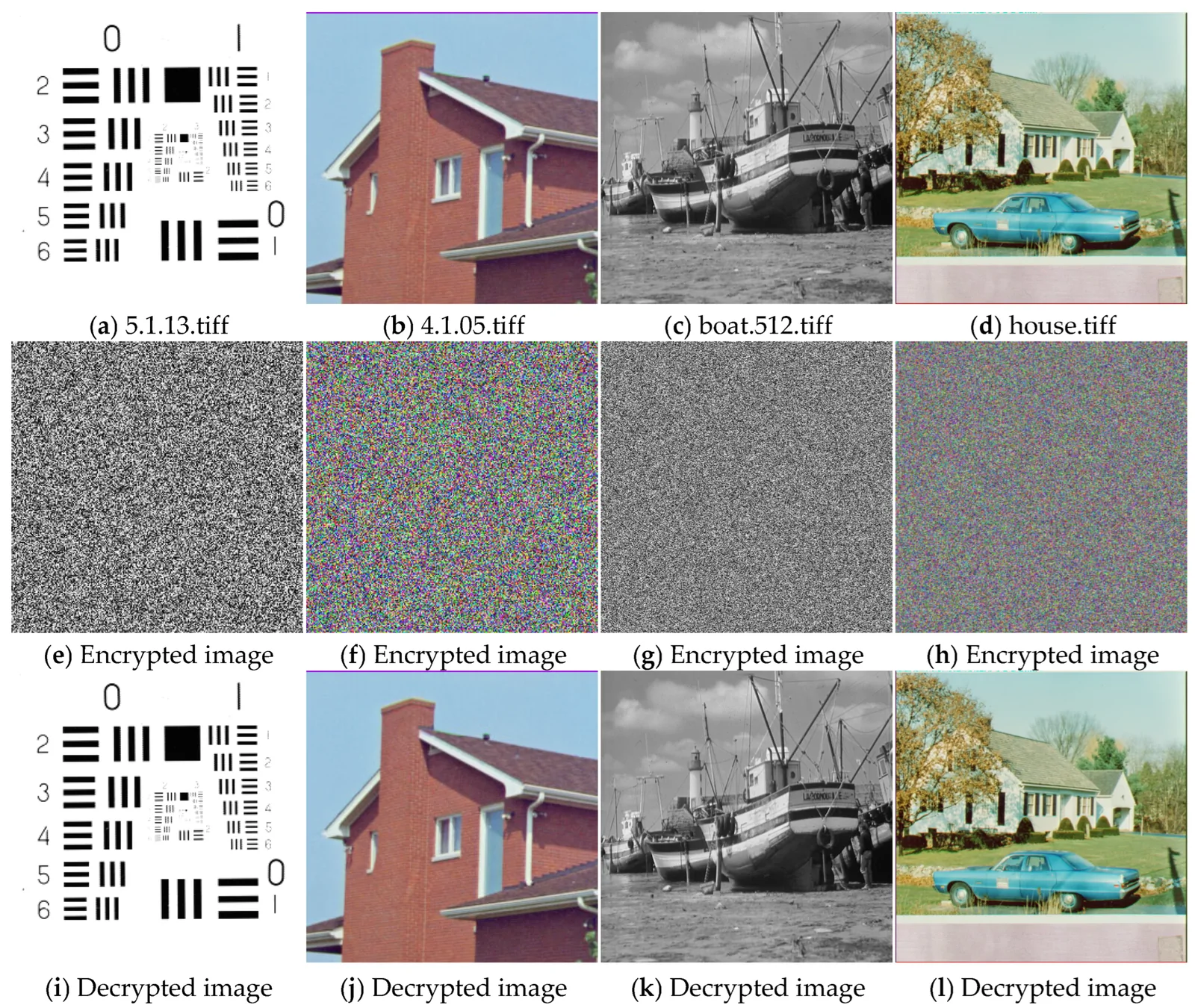
Plaintext images, encrypted images, and decrypted images of representative test images.

As shown in Figure 12a–d, Figure 12a,b have a size of 256 × 256, while Figure 12c,d have a size of 512 × 512. Figure 12a,c are grayscale images, whereas Figure 12b,d are color images. The ciphertext images in Figure 12e–h exhibit noise-like patterns and reveal no plaintext information. The decrypted images in Figure 12i–l are identical to the original images.

### 4.2. Key Space Analysis

A secure encryption algorithm requires a sufficiently large key space to ensure that the system cannot be broken by traversing all possible keys. Generally, it is believed that if the key space is larger than 2^100^, the encryption scheme can effectively resist exhaustive attacks. In the proposed algorithm, the secret key information includes the user master key and the session parameters generated from the mixed hash, namely the initial values $x_{0}$ and $y_{0}$, the control parameters μ and β, and the transient iteration number T. Since $x_{0}$, $y_{0}$, μ and β are real-valued parameters, if the calculation precision of the operating environment is 10^−15^, then the key space contributed by these four parameters alone is no less than 10^15×4^ ≈ 2^199^. Furthermore, the transient iteration number T ranges from 1500 to 2999, which provides 1500 additional possible states. Therefore, the total key space of the proposed encryption scheme is at least 1500 × 10^60^ ≈ 2^209^. Hence, the proposed algorithm can effectively resist exhaustive attacks.

### 4.3. Histogram Analysis

Histogram analysis is commonly used to evaluate whether the encrypted image still preserves visible statistical characteristics of the plaintext image. For a secure image encryption scheme, the histogram of the ciphertext image should be significantly different from that of the plaintext image and should be approximately uniform.

**Figure 13:**
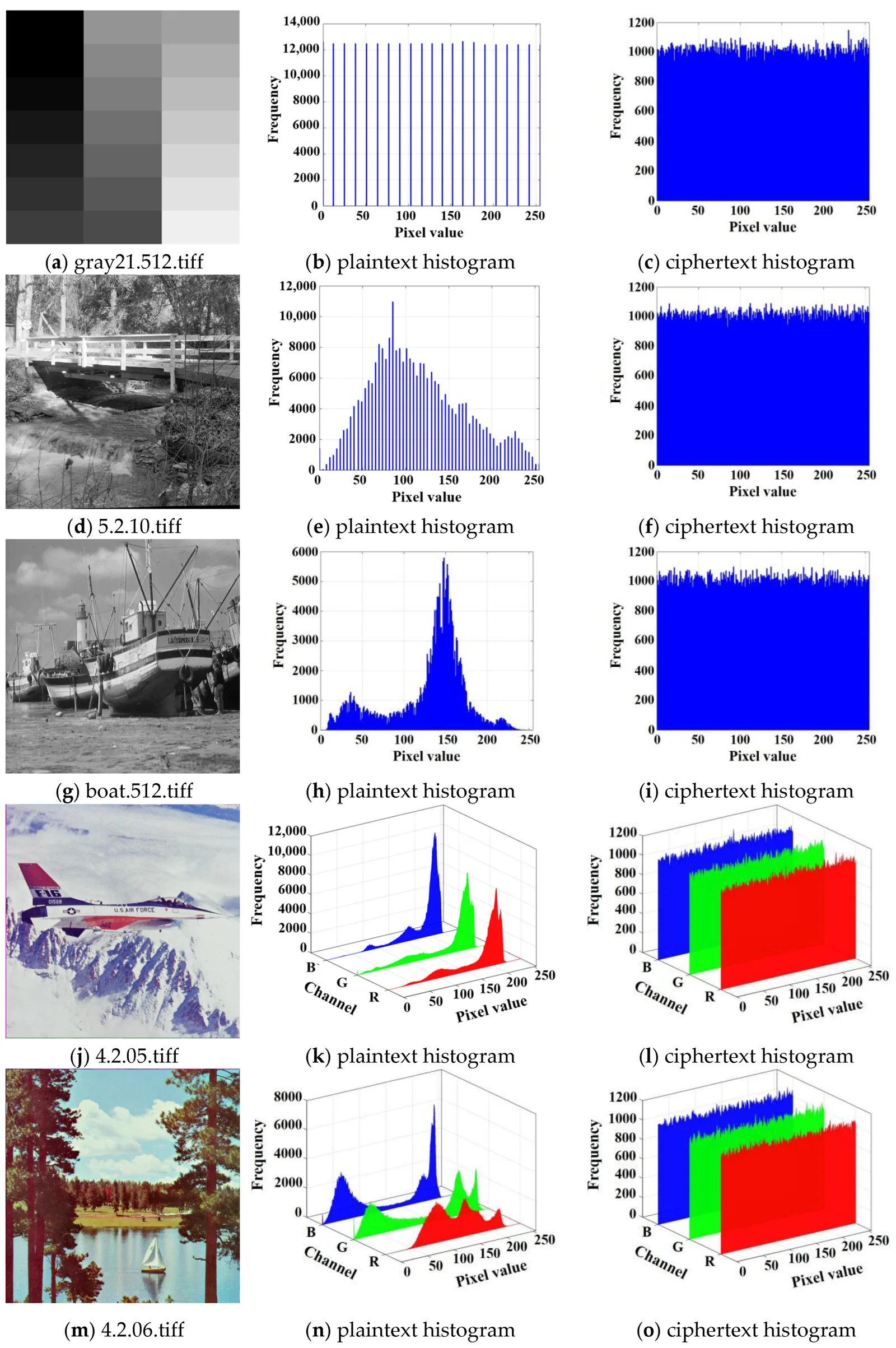

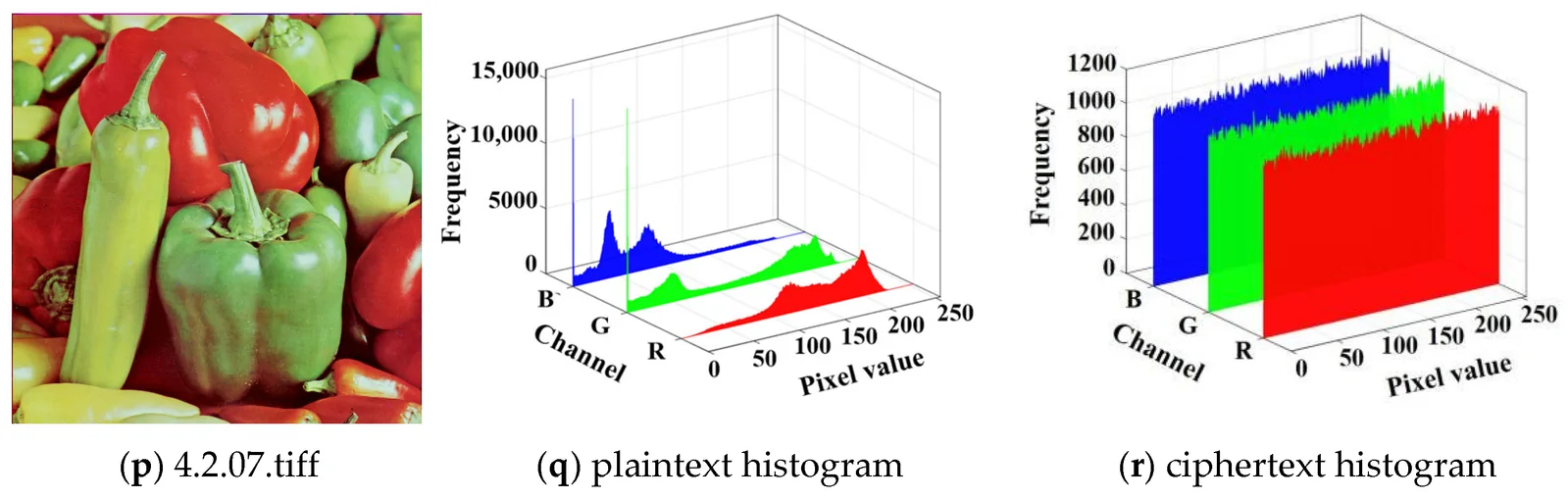
Histogram comparison between plaintext and ciphertext images.

The histogram comparison results are shown in Figure 13. Figure 13a,d,g are grayscale, while Figure 13j,m,p are color images. It can be observed from Figure 13 that the plaintext histograms preserve the original intensity distribution of the natural images, whereas the ciphertext histograms become much flatter and more uniform after encryption. For RGB images, Figure 13 also shows that the ciphertext histograms of the R, G, and B channels all exhibit near-uniform distributions, demonstrating that the proposed method can effectively suppress channel-wise statistical leakage.

### 4.4. Correlation Analysis of Adjacent Pixels

Natural images usually exhibit strong correlations among adjacent pixels in the horizontal, vertical, and diagonal directions. After encryption, these correlations should be significantly reduced. The correlation coefficient is calculated by

(37) $$r_{xy}=\frac{Cov(x,y)}{\sqrt{D(x)}\sqrt{D(y)}},$$

where

(38) $$Cov(x,y)=\frac{1}{N_{s}}\sum_{i=1}^{N_{s}}\left(x_{i}-\bar{x}\right)(y_{i}-\bar{y}),$$

(39) $$D(x)=\frac{1}{N_{s}}\sum_{i=1}^{N_{s}}{\left(x_{i}-\bar{x}\right)}^{2},\;\;D(y)=\frac{1}{N_{s}}\sum_{i=1}^{N_{s}}{\left(y_{i}-\bar{y}\right)}^{2}$$

**Figure 14:**
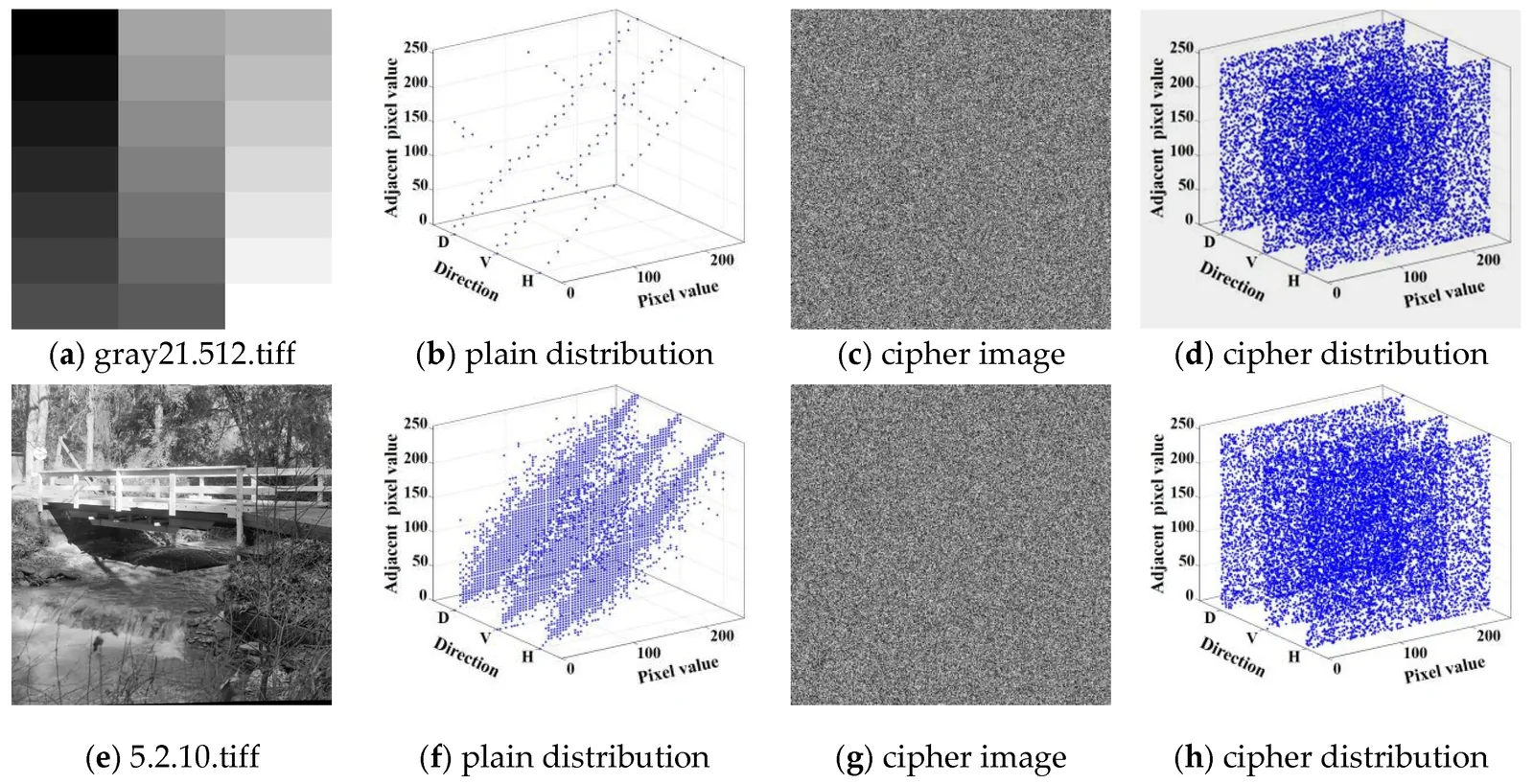

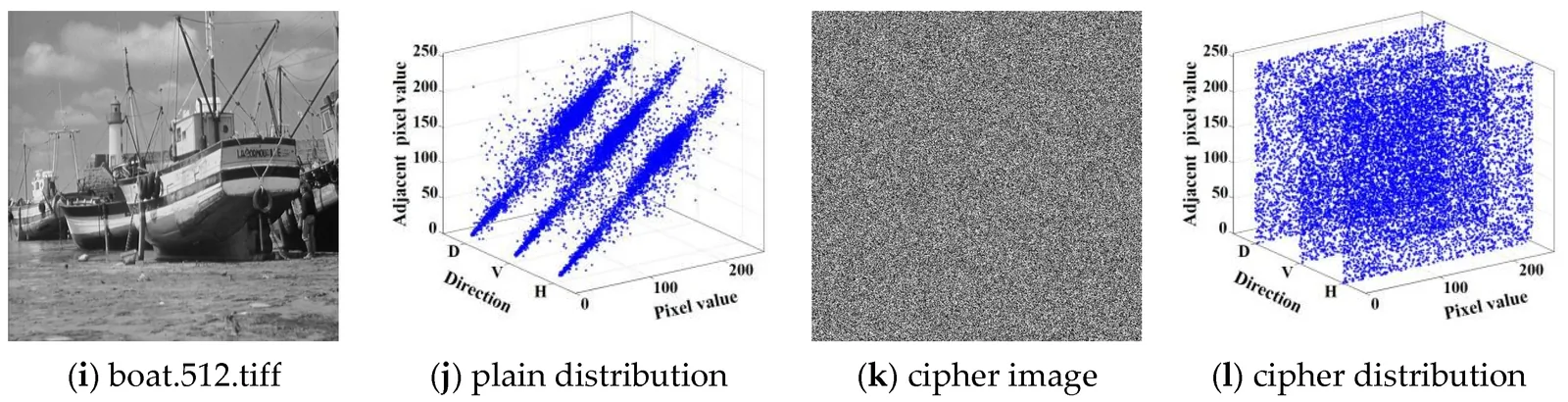
Adjacent-pixel distribution plots of plaintext and ciphertext grayscale images in the horizontal (H), vertical (V), and diagonal (D) directions.

**Figure 15:**
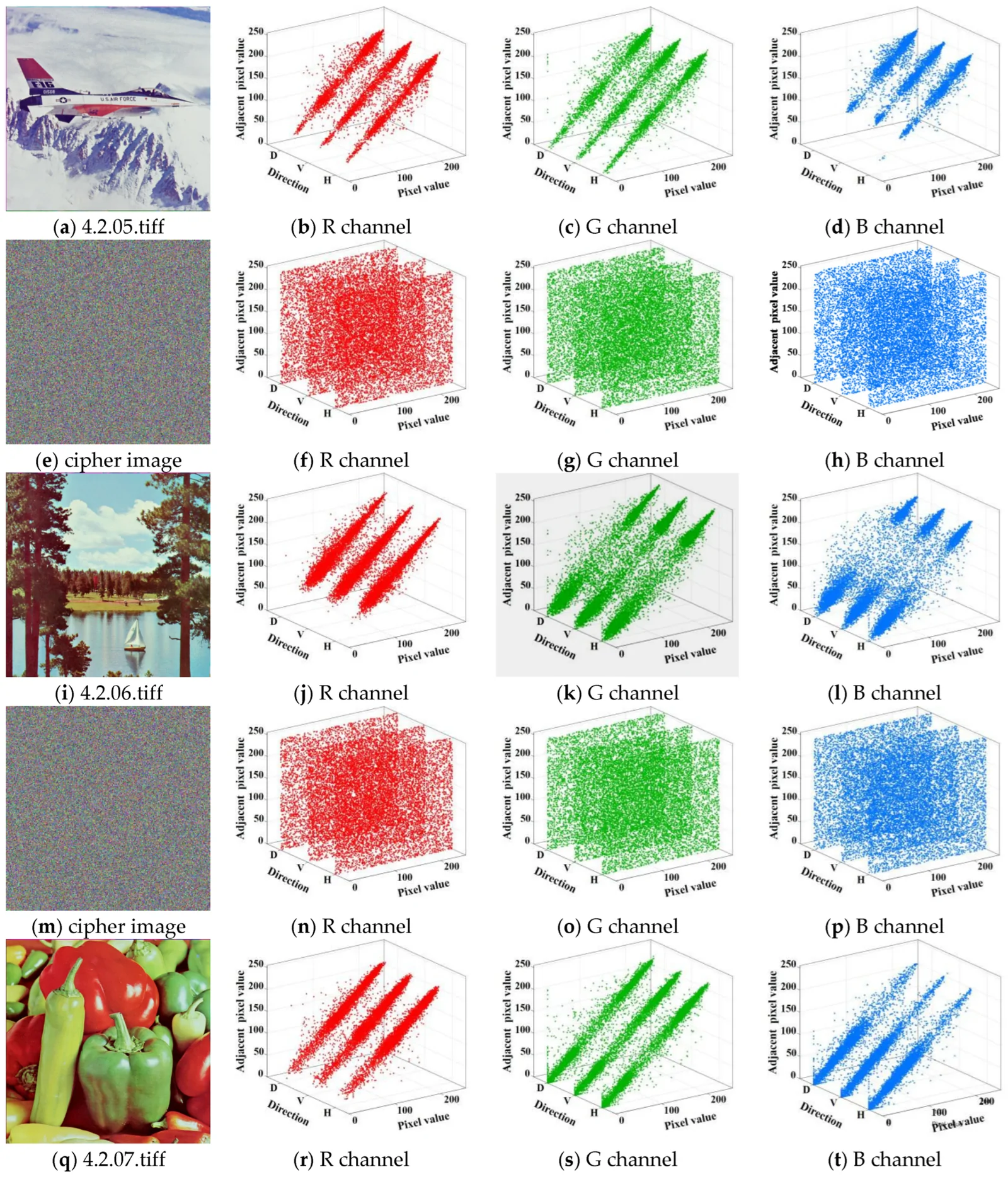

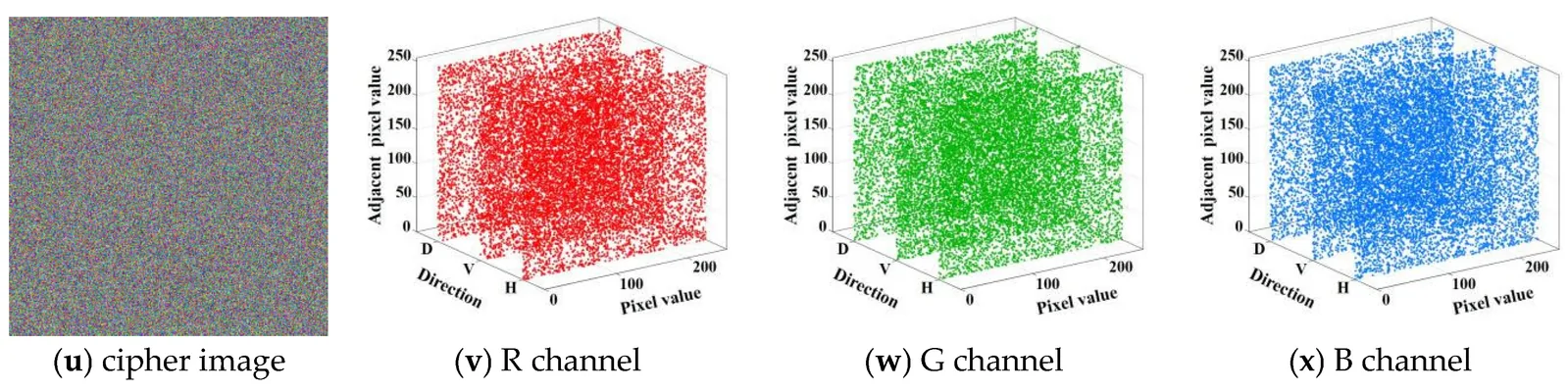
Adjacent-pixel distribution plots of plaintext and ciphertext color images in the horizontal (H), vertical (V), and diagonal (D) directions.

Here, $x_{i}$ and $y_{i}$ denote two adjacent pixel values, $\bar{x}$ and $\bar{y}$ are their mean values, and $N_{s}$ is the number of sampled adjacent pixel pairs. The adjacent-pixel distribution plots are shown in Figure 14 and Figure 15, and the corresponding correlation coefficients are summarized in Table 3. As observed from the distribution plots, adjacent pixels in the plaintext images are mainly concentrated along the diagonal, reflecting strong local correlation, whereas the ciphertext pixels are scattered over a much wider range. The numerical results in Table 3 are consistent with this observation: the plaintext correlation coefficients are close to 1, while those of the ciphertext images in the horizontal, vertical, and diagonal directions are close to 0.

### 4.5. Resistance to Chosen-Plaintext Attack

**Figure 16:**
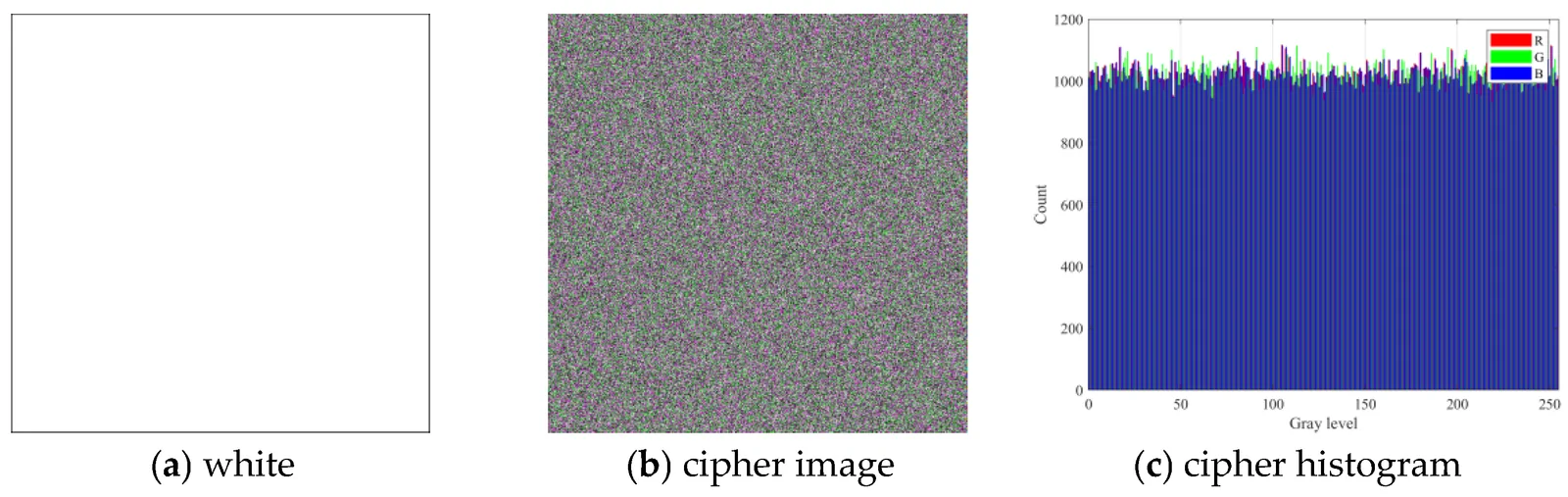

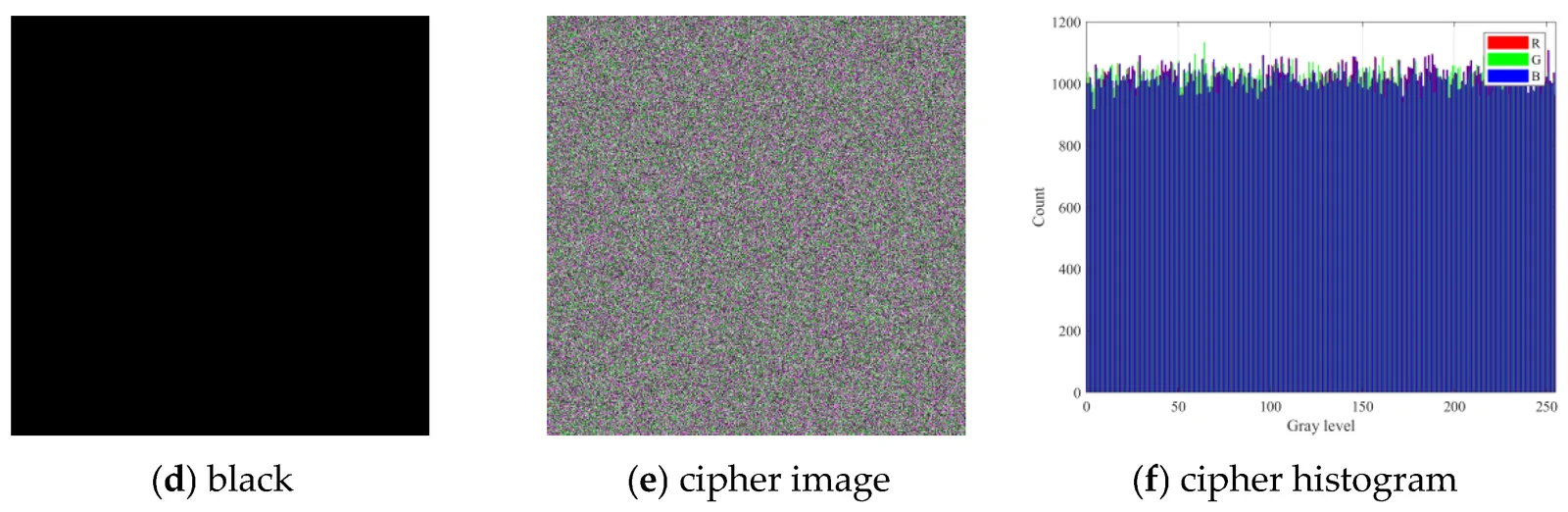
Chosen-plaintext test results for all-white and all-black RGB images.

To assess the resistance of the proposed scheme to chosen-plaintext attacks, two extreme RGB plaintext images, an all-white image and an all-black image, were encrypted using the same secret key. As shown in Figure 16, the ciphertext images exhibit noise-like appearances and do not reveal visible information from the original plaintexts. In addition, the corresponding histograms are approximately uniform, indicating that the statistical characteristics of these highly regular plaintext images are effectively concealed. These results suggest that the proposed scheme has resistance to chosen-plaintext attacks.

### 4.6. Information Entropy Analysis

Information entropy is used to measure the uncertainty of pixel values in an image. For an 8-bit image, the ideal entropy is 8. It is calculated by

(40) $$H=-\sum_{i=0}^{255}p(i)\log_{2}p(i),$$

where $p(i)=n_{i}/N$ denotes the empirical probability of gray level i, $n_{i}$ is the number of pixels with intensity i, and N is the total number of pixels in the evaluated image or color channel. The information entropy results are listed in Table 4. As shown in Table 4, the entropy values of the ciphertext images are close to the ideal value of 8 for both grayscale images and RGB components, indicating that the encrypted images have nearly uniform intensity distributions. This suggests that the statistical characteristics of the plaintext images are effectively concealed after encryption.

Furthermore, Table 5 compares the proposed scheme with several existing image encryption algorithms in terms of information entropy. The comparison results show that the proposed scheme achieves competitive entropy values, further demonstrating its ability to reduce statistical redundancy and resist probability-distribution-based statistical attacks.

### 4.7. Differential Attack Analysis

A secure image encryption scheme should be highly sensitive to tiny changes in the plaintext image. To evaluate the resistance to differential attacks, two widely used metrics, namely the Number of Pixel Change Rate (NPCR) and the Unified Average Changing Intensity (UACI), are adopted. Suppose $C_{1}$ and $C_{2}$ are the ciphertext images obtained by encrypting two plaintext images that differ only in one pixel. NPCR is defined as

(41) $$NPCR=\frac{1}{L}\sum_{i=1}^{L}D_{i}\times 100\%,$$

where

(42) $$D_{i}=\left\{\begin{array}{c} 0,C_{1}(i)=C_{2}(i),\\ 1,C_{1}(i)\neq C_{2}(i). \end{array}\right.$$

UACI is defined as

(43) $$UACI=\frac{1}{255L}\sum_{i=1}^{L}\left|C_{1}(i)-C_{2}(i)\right|\times 100\%.$$

As shown in Table 6, the obtained NPCR and UACI values are close to their theoretical ideal values of 99.6094% and 33.4635%, respectively, indicating that a tiny modification in the plaintext image can propagate rapidly throughout the ciphertext image. Furthermore, the comparison with other algorithms in Table 7 shows that the proposed scheme achieves competitive differential performance, confirming its high plaintext sensitivity and strong resistance to differential attacks.

### 4.8. Robustness Analysis

**Figure 17:**
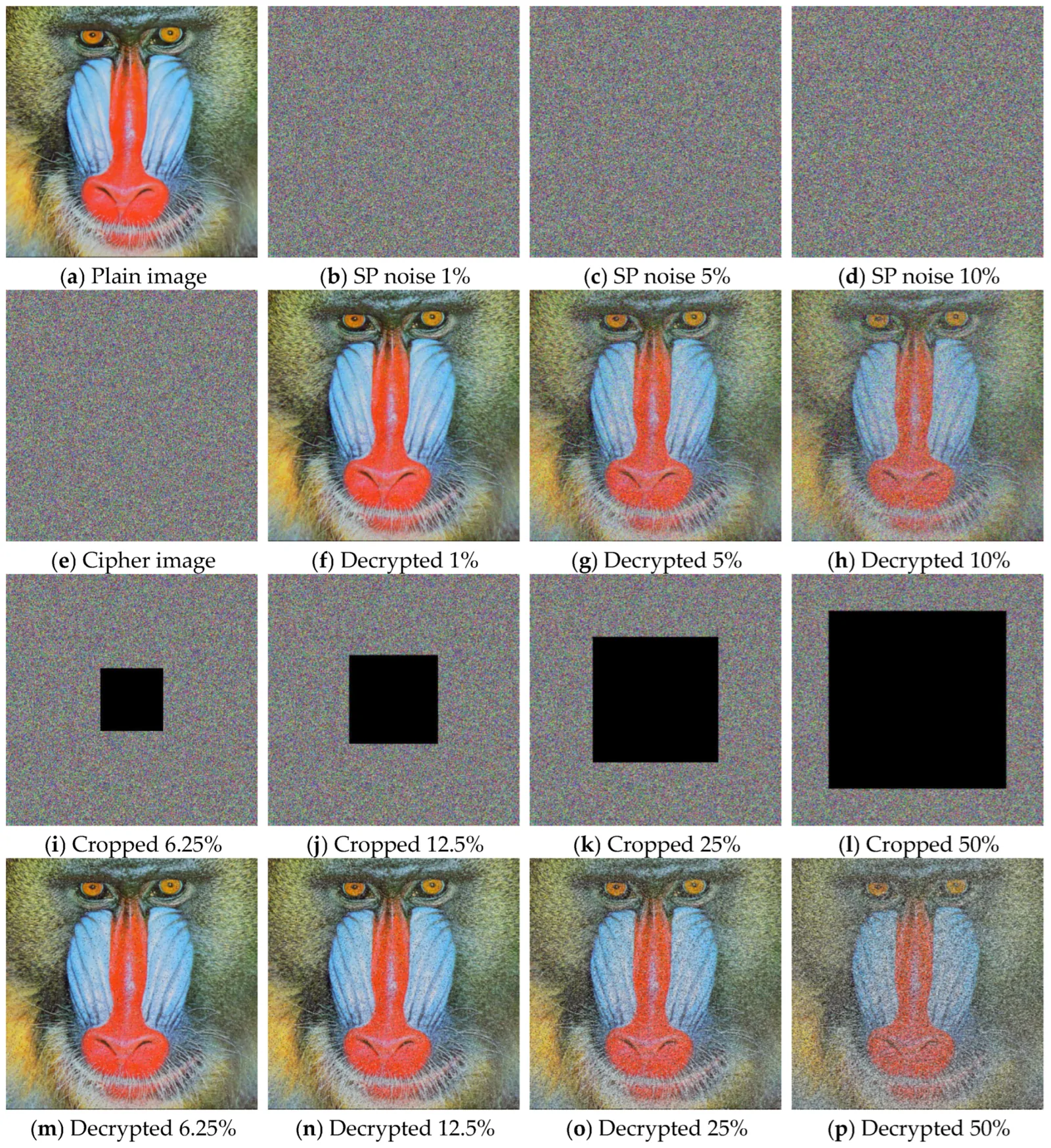
Decryption results under salt-and-pepper noise and cropping attacks.

To evaluate the robustness of the proposed encryption scheme against noise interference and data loss, the Mandrill image was selected as the test image. Salt-and-pepper noise with densities of 1%, 5%, and 10% was added to the ciphertext image. In addition, cropping attacks with ratios of 6.25%, 12.5%, 25%, and 50% were applied to the ciphertext image to further examine the error tolerance of the algorithm. The corresponding results are shown in Figure 17. It can be observed that, under mild noise contamination or limited cropping, the decrypted images still retain recognizable visual information. As the noise density or cropping ratio increases, the quality of the recovered images gradually degrades, but partial image content can still be identified. Peak Signal-to-Noise Ratio (PSNR) [29] is calculated between the original plaintext image and each decrypted image to quantitatively assess the recovered image quality. The results under different salt-and-pepper noise densities and cropping ratios are presented in Table 8.

### 4.9. Computational Complexity and Efficiency Analysis

The computational complexity of the proposed scheme mainly comes from three parts: plaintext-related session-key generation, concentric-ring permutation, and enhanced hybrid diffusion. The session-key generation stage involves hash computation and chaotic sequence initialization, whose cost is approximately linear with respect to the image size. The concentric-ring permutation stage includes ring partition, index sorting, cyclic shifting, and balanced regrouping. Since sorting operations dominate this stage, its computational complexity is approximately O(LlogL). The enhanced hybrid diffusion stage performs multiple forward and backward scans over the image sequence, and therefore its complexity is O(L).

The practical efficiency results are summarized in Table 9. As listed in Table 9, the proposed scheme achieves complete encryption and decryption within acceptable time for representative RGB images, demonstrating that it is computationally feasible for secure image transmission applications. Table 10 compares the average encryption time of the proposed scheme with several recent image encryption algorithms for 512 × 512 color images. The proposed method achieves an average encryption time of 0.4001 s, which is lower than most of the compared schemes, demonstrating its good computational efficiency.

## 5. Conclusions and Outlooks

An image encryption algorithm based on the proposed 2D-FSLM is developed in this paper. By combining plaintext-related session-key generation, concentric-ring permutation, RGB channel permutation, and enhanced hybrid diffusion, the proposed scheme improves key sensitivity, spatial scrambling, inter-channel confusion, and nonlinear diffusion capability. Comprehensive evaluations demonstrate that the algorithm can effectively conceal visual information, suppress statistical leakage and adjacent-pixel correlation, and resist brute-force, statistical, differential, and chosen-plaintext attacks. In addition, it is applicable to both grayscale and RGB images, providing an effective encryption solution for secure image storage and transmission.

In future work, the proposed encryption framework will be further extended to video encryption, medical image protection, and remote sensing image security. Moreover, the integration of image encryption with authentication, integrity verification, and secure transmission protocols will be explored to further enhance its robustness and applicability in real-world secure communication scenarios.

## Figures and Tables

**Table 1:** Definitions of the comparison chaotic systems.

System	Mathematical Definition	Control Parameters
2D-LCCCM	$\begin{array}{c} x_{n+1}=\cos(\pi^{2}(4\mu x_{n}(1-x_{n})+\beta y_{n}(1-y_{n}^{2}))+\frac{\pi }{2})\\ y_{n+1}=\cos(\pi^{2}(4\mu y_{n}(1-y_{n})+\beta x_{n}(1-x_{n+1}^{2}))+\frac{\pi }{2}) \end{array}$	μ,β
2D-SCLM	$\begin{array}{l} x_{n+1}=\sin(\pi ((x_{n}+3)\mu \sin(\pi x_{n})+\beta \sin(\pi y_{n})))\\ y_{n+1}=\sin(\pi ((y_{n}+3)\mu \sin(\pi x_{n})+\beta \sin(\pi x_{n}))) \end{array}$	μ,β
2D-SLEM	$\begin{array}{l} x_{n+1}=\mathrm{mod}(1000\sin(\mu x_{n}(1-y_{n})),1)\\ y_{n+1}=\mathrm{mod}(e^{x_{n}}(1-\sin(\beta y_{n})),1) \end{array}$	μ,β

**Table 2:** NIST SP 800-22 Rev 1a test results of the X and Y binary sequences generated by the proposed 2D-FSLM.

Test Family	*p*-Value (X Sequence)	Result	*p*-Value (Y Sequence)	Result
Frequency	0.085587	Passed	0.994250	Passed
Block Frequency	0.062821	Passed	0.202268	Passed
Cumulative Sums	0.851383	Passed	0.883171	Passed
Runs	0.319084	Passed	0.678686	Passed
Longest Run	0.075719	Passed	0.474986	Passed
Rank	0.350485	Passed	0.759756	Passed
FFT	0.657933	Passed	0.637119	Passed
NonOverlapping Template	0.045675	Passed	0.020548	Passed
Overlapping Template	0.191687	Passed	0.897763	Passed
Universal	0.534146	Passed	0.181557	Passed
Approximate Entropy	0.236810	Passed	0.102526	Passed
Random Excursions	0.035174	Passed	0.090936	Passed
Random Excursions Variant	0.023545	Passed	0.074177	Passed
Serial	0.171867	Passed	0.202268	Passed
Linear Complexity	0.474986	Passed	0.494392	Passed

**Table 3:** Correlation coefficients of adjacent pixels in horizontal, vertical, and diagonal directions for plaintext and ciphertext images.

Image	Component	Plain			Cipher		
		Horizontal	Vertical	Diagonal	Horizontal	Vertical	Diagonal
gray21.512.tiff	gray	0.997662	0.999870	0.996866	0.030877	−0.003563	−0.013514
5.2.10.tiff	gray	0.939611	0.927805	0.909931	−0.011123	0.004540	−0.014732
boat.512.tiff	gray	0.935463	0.972580	0.923772	0.006738	−0.000785	0.010655
4.2.05.tiff	R	0.972638	0.956813	0.934330	−0.000260	−0.001485	−0.000693
	G	0.957784	0.967753	0.932590	−0.000966	0.005211	0.002169
	B	0.963978	0.935321	0.914579	0.000497	−0.001246	−0.000324
4.2.06.tiff	R	0.955791	0.954103	0.941990	−0.002246	0.001117	0.000016
	G	0.971488	0.966258	0.952957	0.000898	0.004209	−0.001648
	B	0.971038	0.969376	0.952958	0.001087	−0.003979	0.000957
4.2.07.tiff	R	0.963525	0.966337	0.956377	0.000257	−0.003328	−0.001337
	G	0.981118	0.981774	0.968658	−0.000229	0.000821	0.001671
	B	0.966517	0.966425	0.947794	−0.000046	−0.000765	0.001515

**Table 4:** Information entropy values of plaintext and ciphertext images.

Image	Component	Plain	Cipher
gray21.512.tiff	Gray	4.3923	7.9993
5.2.10.tiff	Gray	5.7056	7.9993
boat.512.tiff	Gray	7.1914	7.9993
4.2.05.tiff	R	6.7178	7.9993
	G	6.7990	7.9992
	B	6.2138	7.9993
4.2.06.tiff	R	7.3124	7.9992
	G	7.6429	7.9993
	B	7.2136	7.9994
4.2.07.tiff	R	7.3388	7.9994
	G	7.4963	7.9991
	B	7.0583	7.9993

**Table 5:** Comparison Results of Information Entropy.

Image	Mansoor and Parah (2023) [41]	Rezaei et al. (2023) [42]	Li et al. (2025) [43]	L. Wu et al. (2026) [19]	Proposed
4.2.03.tiff	7.9972	7.9976	7.9986	7.9985	7.9993
4.2.05.tiff	7.9971	7.9987	7.9993	7.9972	7.9993
4.2.07.tiff	7.9972	7.9973	7.9981	7.9980	7.9993

**Table 6:** NPCR and UACI results of the proposed encryption scheme.

Image	Component	NPCR (%)	UACI (%)
gray21.512.tiff	Gray	99.5842	33.4152
5.2.10.tiff	Gray	99.5941	33.3776
boat.512.tiff	Gray	99.5983	33.5193
4.2.05.tiff	R	99.6239	33.4488
	G	99.6334	33.5350
	B	99.6021	33.4395
4.2.06.tiff	R	99.6128	33.4833
	G	99.5892	33.3885
	B	99.5991	33.4959
4.2.07.tiff	R	99.6029	33.4211
	G	99.5922	33.4484
	B	99.6284	33.4386

**Table 7:** Comparison of NPCR and UACI with Other Algorithms.

Image	Metric	Wang et al. (2022) [44]	Fan et al. (2023) [45]	Zhang et al. (2024) [46]	Xu et al. (2026) [20]	Proposed
4.2.03.tiff	NPCR (%)	99.6178	99.6115	99.6037	99.5937	99.6076
	UACI (%)	33.5232	33.4526	33.4362	33.3482	33.4577
4.2.05.tiff	NPCR (%)	99.7025	99.6103	99.6029	99.6890	99.6198
	UACI (%)	33.1340	33.4754	33.4287	33.4800	33.4744
4.2.06.tiff	NPCR (%)	99.5491	99.6081	99.6056	99.6206	99.6004
	UACI (%)	33.6372	33.4460	33.4204	33.3998	33.4559
4.2.07.tiff	NPCR (%)	99.6109	99.6063	99.6037	99.5906	99.6078
	UACI (%)	33.7657	33.4729	33.4246	33.4174	33.4360

**Table 8:** Quantitative evaluation of decrypted images under noise and cropping attacks.

Attack Type	Attack Level	R-Channel PSNR (dB)	G-Channel PSNR (dB)	B-Channel PSNR (dB)
Salt-and-pepper noise	1%	21.893	22.492	21.538
	5%	15.367	15.811	14.942
	10%	12.706	13.2	12.345
Cropping attack	6.25%	20.717	21.164	20.291
	12.5%	17.73	18.202	17.324
	25%	14.746	15.211	14.341
	50%	11.722	12.195	11.322

**Table 9:** Encryption time of the proposed scheme for 512 × 512 color images.

Image	Permutation (s)	Diffusion (s)	Total (s)
4.2.01.tiff	0.1835	0.1078	0.4018
4.2.03.tiff	0.1803	0.1078	0.3989
4.2.05.tiff	0.1819	0.1080	0.4017
4.2.06.tiff	0.1806	0.1076	0.3993
4.2.07.tiff	0.1816	0.1076	0.3999
house.tiff	0.1814	0.1074	0.3992
Average	0.1816	0.1077	0.4001

**Table 10:** Average encryption time comparison with recent image encryption algorithms.

Algorithms	Software	CPU	Average Encryption Time (s)
Zhang et al. (2024) [46]	MATLAB R2023a	11th Gen Intel Core i7-11800H CPU @ 2.30 GHz	0.6385
Xin et al. (2023) [47]	MATLAB R2019a	Intel(R) Core(TM) i7-10700, CPU @ 2.90GHz	0.8616
Koulouh et al. (2025) [48]	Python 3.12	Intel(R) Core (TM) i7-4600U CPU @ 2.10 GHz (2.69 GHz)	0.4454
The proposed	MATLAB R2024b	13th Gen Intel Core i7-13650HX @ 2.60 GHz	0.4001

## Data Availability

The raw data supporting the conclusions of this article will be made available by the authors on request.
